# Rapid G4 Ligand Screening Through Spectral Changes Using HT-SRCD with Minimal Material

**DOI:** 10.3390/molecules30163322

**Published:** 2025-08-08

**Authors:** Martina Rotondo, Claudia Honisch, Pietro Spanu, Fausta Ulgheri, Giovanni Loriga, Andrea Beccu, Rohanah Hussain, Barbara Biondi, Paolo Ruzza, Giuliano Siligardi

**Affiliations:** 1 Department of Biology, University of Naples Federico II, 80126 Napoli, Italy; martina.rotondo@unina.it; 2 Padova Unit, Institute of Biomolecular Chemistry of CNR, 35131 Padova, Italy; claudia.honisch@cnr.it (C.H.); barbara.biondi@cnr.it (B.B.); 3 Sassari Unit, Institute of Biomolecular Chemistry of CNR, 07040 Sassari, Italy; pietro.spanu@cnr.it (P.S.); fausta.ulgheri@cnr.it (F.U.); giovanni.loriga@cnr.it (G.L.); andrea.beccu@cnr.it (A.B.); 4 Diamond Light Source, Harwell Science and Innovation Campus, Didcot OX11 0DE, UK; rohanah.hussain@diamond.ac.uk

**Keywords:** high-throughput synchrotron radiation circular dichroism (HT-SRCD), G-quadruplex (G4), drug discovery, ligand screening

## Abstract

The development of molecules that interact with G-quadruplex (G4) sequences requires effective evaluation methods. Several techniques are currently available, including nuclear magnetic resonance (NMR) spectroscopy and X-ray crystallography, surface plasmon resonance (SPR), isothermal titration calorimetry (ITC) and mass spectrometry (MS), fluorescence using FRET-melting, G4-fluorescent intercalator displacement assay (G4-FID) and affinity chromatography. Among these, CD spectroscopy is gaining prominence due to its lower material requirements, faster experimentation and quicker data processing. However, conventional CD methods have limitations, such as higher sample volume required and the inability to handle high-throughput analysis efficiently. The use of synchrotron radiation in high-throughput analysis methods (HT-SRCD) has further advanced the investigation of small-molecule interactions with DNA G4 structures in the presence of various monovalent cations. HT-SRCD offers the capability to analyze multiple samples simultaneously, overcoming the limitations of conventional CD methods. To validate this approach, three biologically relevant G4 sequences—HTelo1, G3T3 and T95-2T—were investigated. Their interactions with a library of small tetrazole-based molecules, synthesized via a four-component Ugi reaction, and with a peptide sequence deriving from RHAU helicases (Rhau25), were evaluated. The results demonstrate that this method not only effectively discriminates between different ligands but also provides valuable insights into the selectivity and the modes of interaction of these ligands with the G4 sequences.

## 1. Introduction

**Figure 1:**
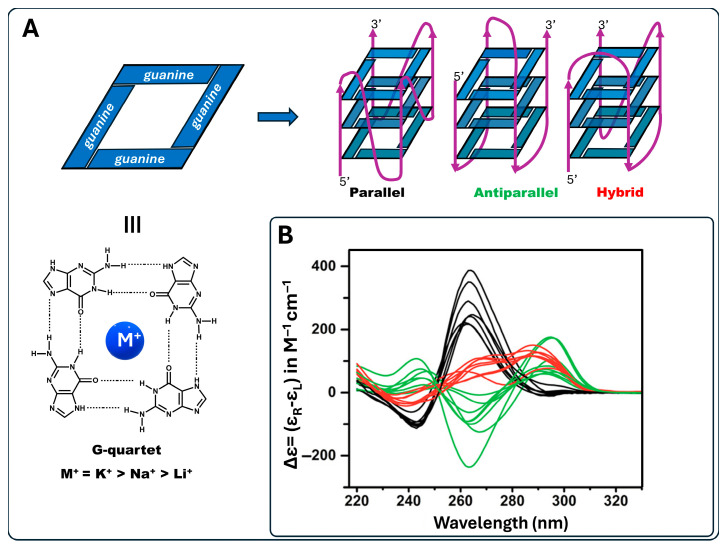
G4 frameworks. (**A**) Structure of a G-quartet consisting of a coplanar structure of four guanines retained by Hoogsteen base pairing and stabilized by a central univalent metal cation (M^+^) and main G4 topologies: Parallel, Antiparallel and Hybrid. (**B**) CD spectra of Parallel (black), Antiparallel (green) and Hybrid (red) topologies redrawn from Villar-Guerra et al. (2018) [23].

G-quadruplex (G4) is a non-canonical secondary structure of nucleic acids that self-folds from sequences with consecutive guanine (G) repeats. Typically, G4 consists of three or more G-quartet (identified as G-tetrad) layers of guanines organized in a coplanar manner. Each G-tetrad is stabilized by Hoogsteen hydrogen bonds and a central monovalent cation, usually K^+^ and Na^+^ ions (Figure 1A) [1,2]. Intracellularly, both sodium and potassium are abundant, but due to their size and the greater dehydration cost of coordinating sodium into the G-tetrad, potassium ions show preferential binding to stabilize G-tetrads [3].

G4s exhibit high polymorphism and can adopt a wide range of structures based on strand molecularity, strand direction, length, and loop composition. The structure may be classified as intramolecular or intermolecular according to the different molecularities. Considering the direction of strands, G4s are categorized as parallel, antiparallel, or hybrid (Figure 1) [4].

G4 structures are identified both in DNA and RNA. Indeed, no structural or physicochemical barrier exists toward forming RNA G4 structures. Moreover, the G4 formation by RNA is more easily facilitated owing to the absence of a competing complementary strand, while DNA G4 formation requires the unwinding of the two strands. The obtained RNA G4 structure has been observed to be more stable than the DNA version [5].

DNA G4s are non-randomly distributed and are primarily located in regions of biological significance, including telomeres [6,7], promoter regions of oncogenes [8,9], and the 5′ untranslated regions of genes [10,11]. This distribution suggests they play an important role in cellular processes such as gene regulation [12], genome stability [4,10], and cellular aging [13]. These characteristics make G4s potential therapeutic targets, particularly in cancer treatment, where aberrant gene expression and genome instability are common [14,15]. Furthermore, the presence of G4 in bacteria and viruses has attracted attention as recent studies have demonstrated the formation and function of G4s in pathogens responsible for serious diseases including *Mycobacterium tuberculosis* [16], *Pseudomonas aeruginosa* [17], *human papilloma virus* (HPV) [18], *human immunodeficiency virus* (HIV) [19,20], and *SARS-CoV-2* [21,22]. Therefore, identifying molecules that can interact with G4 sequences is of fundamental importance in developing new drugs that modulate the activity of G4 sequences.

The binding constant (K_d_) between the ligand and G4 is generally lower than 10^−6^ mol*L^−1^, and the patterns by which ligands can bind to G4 involve π–π stacking interactions with the planar G-tetrad, while the presence of positively charged moieties in the ligand allows interaction with the backbone phosphate groups in grooves and loops [24,25].

Circular dichroism (CD) spectroscopy is a biophysical method widely used to evaluate the secondary structure, folding and binding interaction properties of chiral biopolymers, including nucleic acids. The CD of nucleic acids arises from the coupling of electronic dipole transition of stacked planar aromatic bases of single- or double-stranded DNA folded in a given conformation such as B, C, A and Z for double-stranded DNA or G4 and i-motives for single-stranded DNA to cite the major conformations. Consequently, CD is a powerful tool for studying and characterizing the interaction of G4 with ligands [26]. In contrast to high-resolution methods such as NMR spectroscopy and X-ray crystallography, CD requires far less material and it is much faster [27].

The G4 secondary structures can be identified because of their discriminating CD spectral signatures. A parallel structure is characterized by a positive signal at 264 nm and a negative band at 245 nm, antiparallel G4 shows a positive band at 295 nm and a negative band at 260 nm, while a “hybrid” (or 3 + 1) structure presents positive signals at 295 and 260 nm and a negative band at 245 nm (Figure 1B) [23,26].

**Figure 2:**
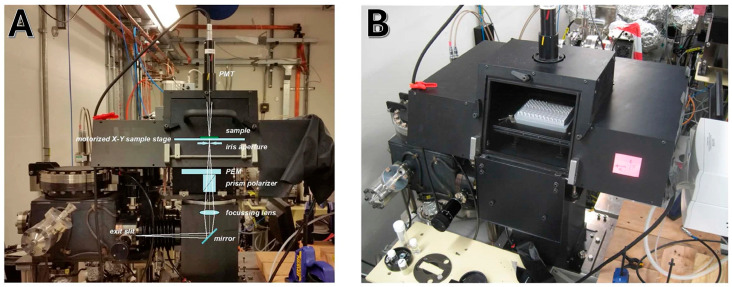
(**A**) Cartoon of the optical elements (exit slit, 45° plane mirror, focusing lens, polarizer prism, PEM, iris, X–Y motorized stage, horizontal sample and PMT detector). The sample is placed on the motorized X–Y stage. (**B**) Picture of the 96-well plate held in the X–Y motorized stage inside the vertical sample chamber unit, redrawn from Hussain et al., 2016 [27].

The use of a CD spectropolarimeter (B23 beamline Diamond Light Source, Didcot, UK) with synchrotron radiation beamlight as the light source provides higher photon flux than commercial instruments, enhancing the signal-to-noise ratio. Diamond B23 beamline generates a highly collimated beamlight that enables CD measurements on small areas of about 1 mm^2^, unattainable with commercial CD instruments and other SRCD beamlines such as Disco (Soleil, France), AU-CD (Astrid2, Denmark), and BL-12 (Hiroshima University, Japan). In this manner, a few microliters of solution can be measured in capillaries, 15 μL with a 0.02 cm pathlength cell and up to 350 μL with a 1 cm pathlength cell of the novel and unique 12-cell strings for high-throughput CD (HT-CD) [27,28,29,30,31,32]. Up to eight strings for the same or different cell pathlengths can be measured as a 96-cell plate (Figure 2), eliminating repeated cell washing compared to using a single cell and reducing time by 80%. Unlike the single-cell autosampling high-throughput method commercialized by Jasco (https://www.jasco.co.uk/cd-htcd.html, accessed on 28 July 2025) and Applied Photophysics (https://www.photophysics.com/product-pages/chirascan-q100/ accessed on 28 July 2025), which use loop-based sample loading that creates dead volume and risks cross-contamination due to strong adherence of some samples, the B23 HT-CD multicell system avoids dead volume and helps minimize cross-contamination between samples.

**Scheme 1:**
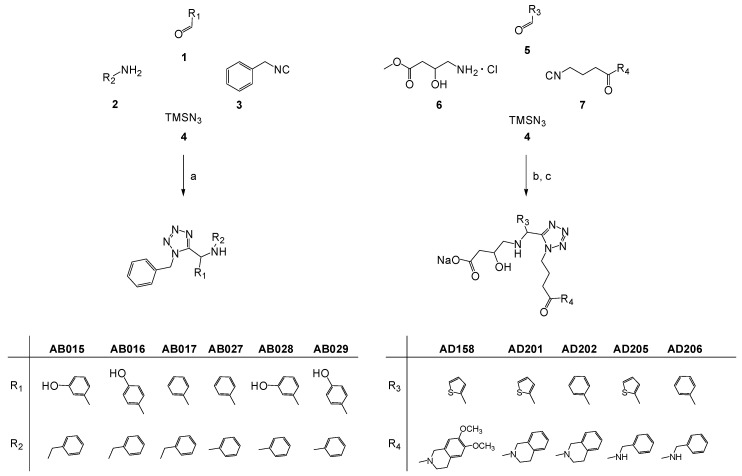
Scheme of 1,5-disubstituted α-amino tetrazole synthesis. Reagents and conditions: (a) CH_3_OH, r.t., 1–5 days (63–80% yields); (b) CH_3_OH, Na_2_SO_4_, Et_3_N, r.t., 5 days (27–45% yields); (c) NaOH, MeOH, r.t., 5 h (64–97% yields).

In this study, HT-SRCD was used to screen a library of small molecules for their binding to G4 structures. Three single-stranded DNA sequences that form G4, HTelo1, G3T3 and T95-2T (Table 1), were studied to characterize their structure and interactions with small molecules (see Scheme 1) in the presence of Na^+^ and K^+^ ions, respectively. The synthesized G4 ligands are significant due to the creation of a novel library of tetrazole derivatives. The interactions with the peptide Rhau25 (Ac-SMHPGHLKGREIGMWYAKKQGQKNK-NH_2_), previously synthesized in our laboratories to interact with the several DNA G-quadruplex sequences [33], were also investigated.

The results obtained highlight the potential of HT-SRCD as a G4 ligand-binding screening tool revealing whether the ligand interaction induces a different G4 topology or changes the CD intensity magnitude compared to the G4 without the ligand.

## 2. Results

### 2.1. Ligand Design and Synthesis

The library of small molecules was designed to meet the common structural requirements of G4 ligands such as an aromatic core to permit π–π stacking interactions with planar G-tetrads. A collection of tetrazole derivatives, easily synthesizable through multicomponent reactions, was chosen to explore the potential of this class of small molecules as G4 ligands and their specificity (Scheme 1).

The library of tetrazole derivatives was synthesized via a one-pot procedure by using a Ugi-tetrazole four-component reaction [34]. 1,5-Disubstituted α-amino tetrazole AB015, AB016, AB017, AB027, AB028 and AB029 were synthesized in good yields, in methanol at room temperature, starting from commercially available benzaldehydes (**1**), amines (**2**), benzyl isocyanide (**3**) and trimethylsilyl azide (**4**). 1,5-Disubstituted α-amino tetrazole AD158, AD201, AD202, AD205 and AD206 were synthesized in methanol at room temperature, starting from commercially available aromatic aldehydes (**5**), trimethylsilyl azide (**4**) and methyl-4-amino-3-hydroxybutanoate hydrochloride as the amine component (**6**), with isocyanides (**7**), both obtained according to our previous reported procedure [35], followed by a basic hydrolysis of the esters obtained (Scheme 1).

### 2.2. HT-SRCD Analysis of G4–Ligand Interactions

**Figure 3:**
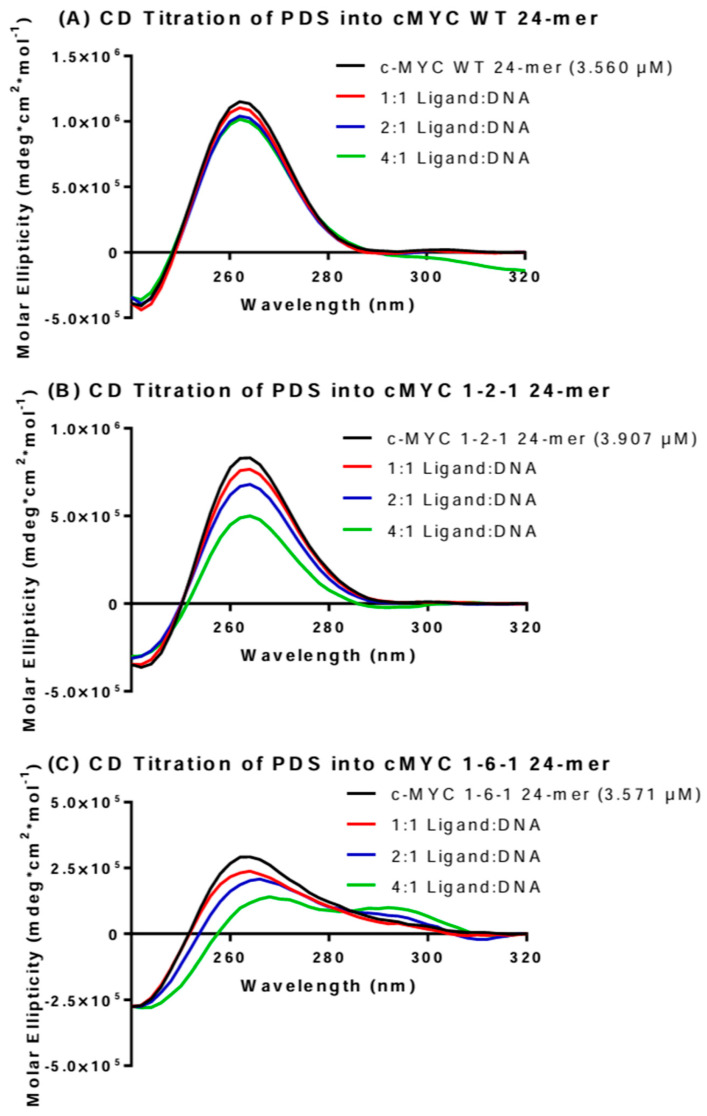
CD spectra of pyridostatin (PDS) titrated into G4 DNA solutions: (**A**) c-MYC WT 24-mer; (**B**) cMYC 1-2-1 24-mer; and (**C**) c-MYC 1-6-1 24-mer measured with the Olis DSM20 spectropolarimeter (redrawn from Z.G. Cuny [36]). The beamline station A used for B23 HT-SRCD data is based on a customized Olis DSM20 monochromator (On-Line Instrument Systems, Inc., Athens, GA, USA) and data acquisition system. This example demonstrates the utility of conformational spectral changes as a validated method for screening ligand-binding interactions in a host–ligand system without the need for immobilization or labeling.

The research examines the role of structural changes in G4s related to protein binding and enzyme inhibition. The screening method described in this manuscript demonstrates effectiveness in identifying G4 ligands with these properties. Additionally, this technique does not require immobilization and is label-free, as previously demonstrated by Cuny [36] using pyridostatin (PDS) (Cambridge Bioscience, CB23 8SQ Cambridge, UK) titrated into G4 DNA solutions (Figure 3).

The interaction of synthesized small molecules of Scheme 1 with G-4 DNA structures was evaluated using HT-SRCD for three distinct G4 sequences: HTelo1 as a model for human telomeric G4 structures; T95-2T mimicking G4 motifs within oncogenic promoters; and G3T3, a synthetic sequence representative of G4 regions involved in gene regulation (Table 1). For HT-SRCD experiments, a custom-made 96-well microplate composed of eight interchangeable strings of twelve HT-CD cells with a pathlength of 1 cm was used. Each cell has a capacity of 350 µL and a window of 6.6 mm in diameter. The presence of two chimneys for sample loading reduces the surface area for sample measurement to approximately 2.5 mm in diameter, requiring the use of a highly collimated beam of a small cross-section, unattainable with a commercial CD instrument but provided by the B23 beamline of the Diamond Light Source synchrotron.

**Figure 4:**
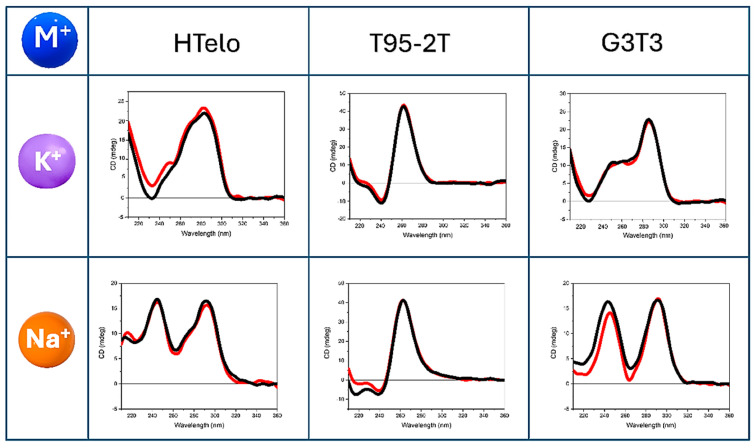
SRCD spectra in the 210–360 nm range of the three different G4 strands alone (black lines) or in the presence of <0.5% MeOH (red lines) in 10 mM Tris-HCl buffer, pH 7.4, in the presence of 70 mM K^+^ or Na^+^ ions, expressed as ellipticity (mdeg), were acquired using a 96-well plate at module A spectropolarimeter.

Stock solutions of the ligands were prepared in MeOH and then diluted to the desired concentration (155 μM final concentration) by adding a buffer containing either potassium or sodium ions, maintaining the alcohol concentration below 0.5%. Preliminary experiments were conducted to evaluate the influence of the small amount of methanol added to the buffer solution on the secondary structure of G4 sequences. As illustrated in Figure 4, CD spectra of G4 in Na^+^ or K^+^ ions were not affected by the addition of <0.5% MeOH.

The assignment of the G4 topologies adopted by HTelo1, T95-2T and G3T3 in both Na^+^ and K^+^ conditions was conducted by comparing the spectral features in Figure 4 to those in Figure 1B.

In 70 mM K^+^, both CD spectra of HTelo1 and G3T3 revealed a dominant contribution of the hybrid topology. In 70 mM Na^+^, the hybrid topology was retained in HTelo1 but changed to antiparallel in G3T3 as its CD shoulder at about 270 nm disappeared. Surprisingly, the CD spectrum of T95-2T was not influenced by the nature of the monovalent cation added to the buffer. In both 70 mM K^+^ and 70 mM Na^+^, the T95-2T CD spectrum showed a strong positive band at approximately 266 nm and a negative band around 245 nm, characteristic of the parallel G4 topology [33,37,38,39,40].

**Figure 5:**
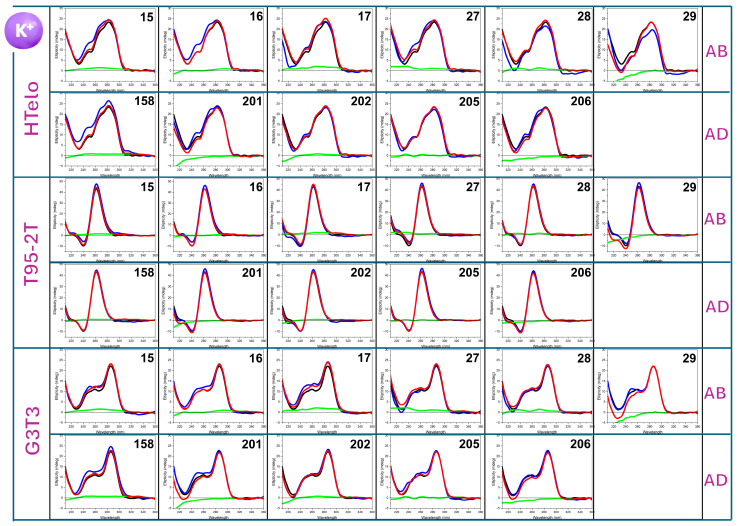
Summary of the SRCD spectra in the 210–360 nm range of G4 sequences (HTelo1, T95-2T, G3T3) in the presence of different ligands (from left to right: AB series = AB015, AB016, AB017, AB027, AB028, AB029; AD series = AD158, AD201, AD202, AD205, AD206) in 10 mM Tris-HCl buffer, pH 7.4, in the presence of 70 mM K^+^ acquired using a 96-well plate at module A. Each box contains the spectrum of the G4 alone (black), the measured (blue) and the calculated (red) spectra of the ligand–G4 complex and the spectrum of the ligand alone (green line) expressed as ellipticity (mdeg).

**Figure 6:**
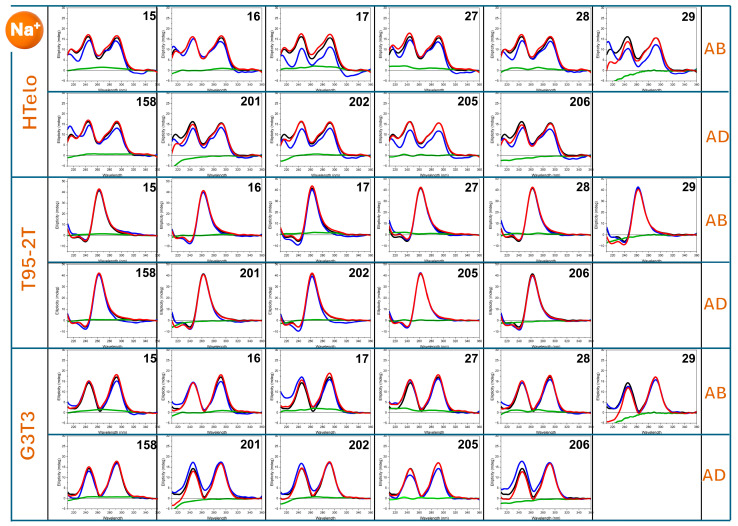
Summary of the SRCD spectra in the 210–360 nm range of G4 sequences (HTelo1, T95-2T, G3T3) in the presence of different ligands (from left to right: AB series = AB015, AB016, AB017, AB027, AB028, AB029; AD series = AD158, AD201, AD202, AD205, AD206) in 10 mM Tris-HCl buffer, pH 7.4, in the presence of 70 mM Na^+^ acquired using a 96-well plate at module A. Each box contains the spectrum of the G4 alone (black), the measured (blue) and the calculated (red) spectra of the ligand–G4 complex and the spectrum of the ligand alone (green line) expressed as ellipticity (mdeg).

The screening of the small molecules listed in Scheme 1 as potential ligands of HTelo1, T95-2T and G3T3 sequences as a function of K^+^ and Na^+^, respectively, carried out with HT-CD using several strings developed at the B23 beamline at Diamond Light Source UK are illustrated in Figure 5 and Figure 6. The degree of CD changes, indicative of ligand binding, observed between the G4 sequences alone and the corresponding solution of G4 with added small molecules was found to be sequence as well as Na^+^ and K^+^ dependent. With regard to the type of G4 sequences used, it was observed that the CD of the T95-2T sequence was much less influenced by the addition of the small molecules, meaning that no molecules bound significantly to T95-2T nor did sodium and potassium promote any difference in the ligand-binding performance. As shown in Figure 5 and Figure 6, a common feature of all the ligands examined was their inability to induce topology changes in the G4 sequences investigated.

**Figure 7:**
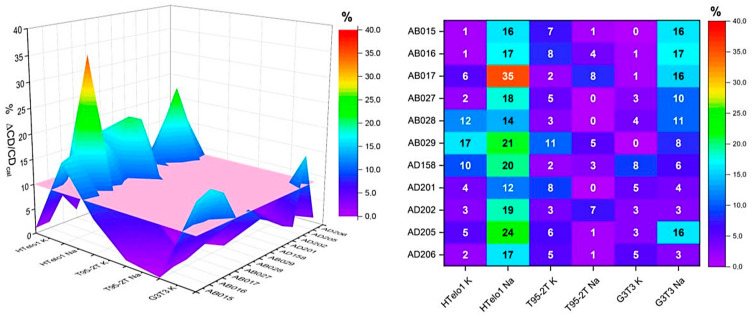
Summary of the percentage (%) change between the measured and calculated dichroic spectrum for each G4–ligand complex in the presence of potassium or sodium ions.

To facilitate the data analysis of Figure 5 and Figure 6, the percentage of variation between the CD spectra of the G4–ligand mixtures determined experimentally and calculated as the sum of the individual contributions from G4 alone and free ligand is reported in Figure 7. The difference between the two spectra was quantified by measuring the absolute value at the wavelength corresponding to the maximum of the CD signal. For G3T3 and HTelo1, which adopt a hybrid topology in the presence of K^+^ ions characterized by two positive CD bands at about 255 and 290 nm, we used the data corresponding to the CD band at these longer wavelengths.

The results summarized in Figure 7 indicate that all the analyzed molecules induce a change greater than 10% in the CD of HTelo1 in the presence of Na^+^. For T95-2T, none of the analyzed molecules induced a significant variation greater than 10% between the measured and calculated CD spectra in Na^+^ or K^+^. G3T3, on the other hand, showed that molecules AB015, AB016, AB017 and AD205 induced a variation greater than 10%, whilst molecules AB027 and AB028 showed a variation of approximately 10%.

These results indicate that sodium ions enable all synthesized compounds to interact with the HTelo1, inducing a conformational change in its structure. However, none of the synthesized molecules induced a significant variation in the CD signal of the T95-2T sequence and only some molecules were able to modify the CD of G3T3. This HT-SRCD screening suggests that these compounds selectively recognize specific G4 structures, particularly the antiparallel forms of HTelo1 and G3T3 in the presence of sodium ions.

In the presence of potassium ions, most of the compounds examined did not induce a significant CD change for the three G4 sequences investigated. Under these conditions, only compounds AB028 and AB029 induced changes greater than 10% between the measured and calculated CD spectrum of HTelo1 (11% and 16%, respectively). It was observed that, in the presence of compound AB029, a significant change also occurred between the measured and calculated CD spectrum for T95-2T, which did not exhibit any conformational change in the presence of sodium ions. Conversely, the G3T3 sequence in the presence of potassium ions did not display any change in the CD upon the addition of the small molecules. This different behavior can be attributed to the different influences of Na^+^ and K^+^ ions on the G4 structures. In the presence of sodium ions, the three G4 sequences exhibited greater conformational flexibility, promoting a more open topology that enhances ligand accessibility allowing multivalent interactions. Conversely, in the presence of potassium ions, the G4s adopted a more compact conformation that restricts ligand accessibility. The influence of potassium ions on the conformation and stability of HTelo1, T95-2T and G3T3 is evident from the analysis of the melting curves obtained in the presence of Na^+^ and K^+^, respectively, showing an increased T*_m_* from Na^+^ to K^+^, ranging from 10 °C for HTelo1 to almost 32 °C for T95-2T (Figure S34 in the Supplementary Material).

**Figure 8:**
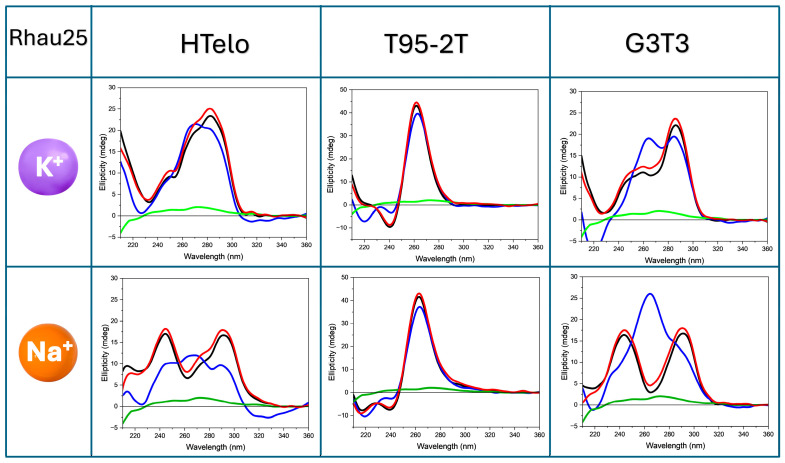
HT-SRCD spectra of G4 sequences in the presence of Rhau25 peptide in 10 mM Tris-HCl buffer, pH 7.4, in the presence of 70 mM K^+^ or Na^+^ ions, acquired using 2 string plates using B23 module A. Each box contains the spectrum of the G4 alone (black), the measured (blue) and the calculated (red) spectra of the ligand–G4 complex and the spectrum of the ligand alone (green line).

The high-throughput SRCD assay’s capability to determine the interaction of the three G4 sequences was also studied with the Rhau25 peptide, previously synthesized and investigated as a G4 ligand by our research group [33]. In the presence of potassium ions, the addition of the Rhau25 peptide did not induce significant CD changes when added to both HTelo1 and T95-2T. Nonetheless, it caused a change in the shoulder present in the CD of G3T3, showing a well-defined positive CD band at about 250 nm in the presence of both Na^+^ and K^+^ (Figure 8).

The replacement of potassium ions with sodium ions demonstrated the ability of Rhau25 to induce conformational changes. In 70 mM Na^+^ the appearance of a positive CD band at about 270 nm for Rhau25-HTelo1 is consistent with a parallel topology contribution on top of the hybrid without the peptide (Figure 8). A similar effect was observed for Rhau25-G3T3 where a stronger positive CD band at 270 nm characteristic of a parallel topology contribution was observed compared to the antiparallel topology without the peptide (Figure 8). For T95-2T the parallel topology was retained with the addition of Rhau25 in both Na^+^ and K^+^ with only a decreased intensity magnitude of the positive CD band at about 265 nm by approximately 15%, corroborating the varying stability displayed by the three G4 sequences in the presence of 70 mM Na^+^ and K^+^, respectively.

## 3. Materials and Methods

All solvents and chemicals were purchased from Sigma Aldrich (Milan, Italy) and used without further purification.

### 3.1. Ligand Synthesis

#### 3.1.1. General MCR Procedure for the Synthesis of 1,5-Disubstituted α-Amino Tetrazoles Series AB 

To a 0.11 M methanol solution of aldehyde **1** (1 equiv.), amine **2** (1 equiv.), benzyl isocyanide **3** (1 equiv.) and TMSN **4** (1 equiv.) were added. The reaction mixture was stirred at room temperature for 1 to 5 days to yield the crude product, which was purified by filtration on a silica gel pad using a 6:4 petroleum ether/EtOAc eluent to give a thick, light-yellow oil with yields ranging from 63% to 80%.

^1^H and ^13^C NMR spectra are reported in the Supplementary Material (Figures S1–S18).

#### 3.1.2. General MCR Procedure for the Synthesis of 1,5-Disubstituted α-Amino Tetrazoles Series AD

To a 0.11 M methanol solution of amine hydrochloride component **6** Lit (1 equiv.), Et_3_N (1 equiv.), Na_2_SO_4_, and after 10 min, the oxo component **5** (1 equiv.) were added. The solution was stirred for 15 min, and then a solution of isocyanide component **7** Lit (1 equiv.) in CH_3_OH (0.44 M) and TMSN **3** (4) (1 equiv.) were added. The reaction mixture was stirred at room temperature for 5 days and then purified by filtration on a silica gel pad by eluting with 9/1 *v*/*v* CH_2_Cl_2_/Et_2_O, then 9/1 *v*/*v* EtOAc/acetone, to give a thick light-yellow oil with yields ranging from 27% to 45%. The 4-MCR product was then solved in CH_3_OH (0.05 M) and treated with NaOH 1M (0.1 M in CH_3_OH). The solution was stirred at room temperature for 5 h, then the solvent was removed under vacuum and the crude product was purified by filtration on a silica gel pad by eluting with EtOAc/acetone 9/1 and CH_2_Cl_2_/CH_3_OH 8/2 to give the desired product, with yields ranging from 64% to 97%. 

^1^H and ^13^C NMR spectra are reported in the Supplementary Material (Figures S19–S33).

### 3.2. Peptide Synthesis

Rhau25 peptide (Ac-SMHPGHLKGREIGMWYAKKQGQKNK-NH_2_) was obtained by automated solid phase peptide synthesis (SPPS) using the Fmoc/HBTU chemistry approach by means of a Biotage^®^ Syro Wave™ synthesizer (Biotage AB, Uppsala, Sweden) controlled by Syro XP peptide software. Acetylation was obtained by treatment with acetic anhydride, and the cleavage from the Rink-amide resin was performed by treatment with TFA in the presence of TIS and water as scavengers. The peptide was precipitated by ethyl ether and purified by elution on a Dionex Vydac reverse phase C18 300 Å, 10 µ, 22 × 250 mm column using a preparative Shimadzu HPLC system (Kyoto, Japan) equipped with LC-8A pumps, an SLC-8A controller, an SPD-6A spectrophotometric detector and an ERC-3562 ERMA degasser. LC-ESI-MS analyses were performed on an Agilent 1260 Infinity II analytical HPLC system (G7129A vialsampler, G7117C DAD HS and G7111B Quat. Pump) equipped with an Agilent 6130 Quadrupole LC-MS analyzer (Agilent Technologies, Milano, Italy). The calculated mass was 2951.41 Da.

### 3.3. Preparation of G-Quadruplex Sequences

Synthetic oligonucleotides were purchased from Eurogentech (Seraing, Belgium). The deoxyoligonucleotides were dissolved in ultrapure water (Veolia Water Technologies, Padova, Italy) and the stock solution concentration was determined spectrometrically at 90 °C (ε @ 260 nm = 236,500 for HTelo1; 197,300 for G3T3; 172,600 for T95-2T). Stock solutions were then diluted to the desired concentration to achieve a 4.5 μM solution in 10 mM Tris-HCl buffer, pH 7.4, in the presence of either 70 mM potassium or sodium ions. Solutions were annealed by heating at 90 °C for 10 min and slowly cooling to room temperature.

### 3.4. Preparation of Small-Molecule Ligands

Each ligand was dissolved in methanol to obtain a stock solution of 1 mg/mL. Ligands were then diluted with 10 mM Tris-HCl buffer, pH 7.4, containing either 70 mM Na^+^ or K^+^ ions to get the final desired concentrations (0.155 mM) immediately before use. The final methanol concentration was maintained below 0.5% in all assays.

### 3.5. High-Throughput Synchrotron Radiation Circular Dichroism (HT-SRCD)

HT-SRCD experiments were conducted at module A of the B23 beamline at Diamond Light Source, using a custom setup 96-well microplate designed for high-throughput measurements, which was placed in a vertical sample compartment on a motorized X–Y stage for precise positioning. Briefly: 360 µL of a G4 solution was loaded into a well, and 20 µL of a ligand solution was added to achieve a 1:2 G4/ligand molar ratio. For all wells, the CD spectra were recorded in the 210–360 nm range at 25 °C. Control spectra of G4 alone and ligands alone were also recorded under identical conditions. Measurements were performed by acquiring one scan per well at 39 nm/min, data pitch was 1 nm, bandwidth was 1 nm, using an integration time of 2 s/data point.

SRCD melting experiments were performed in the 5–95 °C range with 10 °C steps and allowing 120 s equilibration time. SRCD spectra were collected on module A of beamline B23 of the Diamond Light Source Ltd. synchrotron facility, Harwell Science and Innovation Campus (Didcot, UK) in the 200–360 nm range in 0.05 cm pathlength quartz cuvettes (Hellma Analytics, Essex SS2 6HZ, UK). Strand concentration was 101 μM in 10 mM Tris-HCl buffer, pH 7.4, in the presence of 70 mM K^+^ or Na^+^ ions.

Collected spectra were processed using OlisWorks (On-Line Instrument Systems, Inc., Athens, GA, USA), CDApps [41] and OriginPro2025 (OriginLab Corporation, Northampton, MA, USA).

## 4. Conclusions

By systematically evaluating the binding affinities and specificities of various ligands under different ionic conditions, we have highlighted the critical role of the ionic environment in modulating G4 conformations and ligand accessibility. This study reveals that sodium ions enhance the conformational flexibility of HTelo1, G3T3 and T95-2T G4 structures, promoting more open topologies that facilitate ligand binding. This insight is crucial for designing ligands that can effectively target G4 structures in sodium-rich cellular environments, potentially leading to more effective therapeutic agents. The study demonstrates the selective binding potential of the tetrazole derivative molecules towards HTelo1 in the presence of Na^+^ rather than K^+^ based on the percentage of CD changes. This selectivity in sodium-rich conditions suggests that such ligands could be tailored to target G4 structures involved in key regulatory processes, thereby improving therapeutic precision and reducing off-target effects. On the other hand, the peptide Rhau25 significantly affects the topologies of HTelo1 and G3T3, more in Na^+^ than K^+^. This study highlights the potential to screen ligands that induce structural changes in G4s. Given the critical role of these structural changes in protein binding and enzyme inhibition, the screening method presented in this manuscript is a powerful tool for identifying G4 ligands with these significant properties.

The impact of our research is multifaceted. Firstly, it advances the understanding of G4–ligand interactions, which is crucial for the development of novel therapeutic agents targeting diseases such as cancer and viral infections. The ability to identify and characterize potential G4 ligands with high precision accelerates the drug discovery process, paving the way for new treatments. Secondly, the high-throughput nature of B23 HT-SRCD positions it as a powerful method for large-scale screening, significantly enhancing the efficiency of identifying promising therapeutic candidates. Lastly, the successful application of HT-SRCD in this study sets a precedent for its broader adoption in structural biology and pharmacology, increasing the visibility of this technique within the scientific community.

In conclusion, the insights gained from this study underscore the importance of considering ionic conditions in the design of G4-targeting ligands. Future research should focus on further exploring the structural dynamics of G4–ligand interactions and optimizing ligand designs to enhance their specificity and efficacy in various biological contexts. HT-SRCD emerges as a groundbreaking approach in the study of G4s, offering unparalleled insights and fostering advancements in therapeutic development. The results of this study not only validate the efficacy of HT-SRCD but also highlight its potential for future applications in drug discovery and beyond.

## Figures and Tables

**Table 1:** G-quadruplex sequence.

Strand	Sequence
HTelo1	5′-TAGGGTTAGGGTTAGGGTTAGGG-3′
T95-2T	5′-TTGGGTGGGTGGGTGGGT-3′
G3T3	5′-GGGTTTGGGTTTGGGTTTGGG-3′

## Data Availability

Data are contained within the article and Supplementary Materials.

## Supplementary Materials

The following supporting information can be downloaded at: https://www.mdpi.com/article/10.3390/molecules30163322/s1, Figure S1. Structure of compound AB15. Figure S2. ^1^H NMR spectrum of compound AB15. Figure S3. ^13^C NMR spectrum of compound AB15. Figure S4. Structure of compound AB16. Figure S5. ^1^H NMR spectrum of compound AB16. Figure S6. ^13^C NMR spectrum of compound AB16. Figure S7. Structure of compound AB17. Figure S8. ^1^H NMR spectrum of compound AB17. Figure S9. ^13^C NMR spectrum of compound AB17. Figure S10. Structure of compound AB27. Figure S11. ^1^H NMR spectrum of compound AB27. Figure S12. ^13^C NMR spectrum of compound AB27. Figure S13. Structure of compound AB28. Figure S14. ^1^H NMR spectrum of compound AB28. Figure S15. ^13^C NMR spectrum of compound AB28. Figure S16. Structure of compound AB29. Figure S17. ^1^H NMR spectrum of compound AB29. Figure S18. ^13^C NMR spectrum of compound AB29. Figure S19. Structure of compound AD158. Figure S20. ^1^H NMR spectrum of compound AD158. Figure S21. ^13^C NMR spectrum of compound AD158. Figure S22. Structure of compound AD201. Figure S23. ^1^H NMR spectrum of compound AD201. Figure S24. ^13^C NMR spectrum of compound AD201. Figure S25. Structure of compound AD202. Figure S26. ^1^H NMR spectrum of compound AD202. Figure S27. ^13^C NMR spectrum of compound AD202. Figure S28. Structure of compound AD205. Figure S29. ^1^H NMR spectrum of compound AD205. Figure S30. ^13^C NMR spectrum of compound AD205. Figure S31. Structure of compound AD206. Figure S32. ^1^H NMR spectrum of compound AD206. Figure S33. ^13^C NMR spectrum of compound AD206. Figure S34. SRCD melting curves for HTelo 1, G3T3, and T95-2T G4s in 10 mM phosphate buffer, pH 7.4, in the presence of 70 mM potassium or sodium ions (indicated). The ellipticity was monitored at the maximum wavelength of each G4 spectrum.
