# Supplementary material for: Rapid G4 Ligand Screening Through Spectral Changes Using HT-SRCD with Minimal Material
Source: Molecules. 2025 Aug 8;30(16):3322. doi: 10.3390/molecules30163322 (PMC12388515; doi:10.3390/molecules30163322)
Supplement: Supplementary file 1 [file molecules-30-03322-s001.zip › molecules-3760440-supplementary.pdf]

# Rapid G4 Ligand Screening Through Spectral Changes Using HT-SRCD with Minimal Material

**Martina Rotondo [1,†], Claudia Honisch [2,†], Pietro Spanu [3], Fausta Ulgheri [3], Giovanni Loriga [3], Andrea Beccu [3], Rohanah Hussain [4], Barbara Biondi [2], Paolo Ruzza [2,*] and Giuliano Siligardi [4,*]**

1 Department of Biology, University of Naples Federico II, 80126 Napoli, Italy
2 Institute of Biomolecular Chemistry of CNR, Padova Unit, 35131 Padova, Italy
3 Institute of Biomolecular Chemistry of CNR, Sassari Unit, 07040 Sassari, Italy
4 Diamond Light Source, Harwell Science and Innovation Campus, Didcot OX11 0DE, UK
* Correspondence: paolo.ruzza@cnr.it (P.R.); giuliano.siligardi@diamond.ac.uk (G.S.)
† These authors contributed equally to this work.

# Supplementary Material

## 1. Material and methods

### *1.1. NMR experiments*

$^1$H and $^{13}$C NMR spectra were acquired on a Bruker Ascend 600 MHz. The chemical shift unit was ppm, and the coupling constant unit was Hz. Proton coupling modes were described as singlet (s), broad singlet (brs), doublet (d), doublet of doublets (dd), triplet (t), and multiplet (m). Column chromatography was performed using 230–400 mesh silica gel. All chemical reagents and solvents were purchased from commercial sources and used without further purification. The purity of the final compounds was determined by NMR spectroscopy, and elemental analysis was in agreement with the proposed structures, with purity ≥95%.

## 2. Ligand characterization

*3-((1-benzyl-1H-tetrazol-5-yl)(benzylamino)methyl)phenol (**AB015**)*

The title compound prepared following the general procedure 1, was obtained as a white solid in 73% yield after 5 days. $^1$H NMR $CDCl_3$ δ 7.31 – 7.22 (m, 4H), 7.18 (d, J = 6.7 Hz, 2H), 7.14 (t, J = 7.8 Hz, 1H), 6.95 (d, J = 7.4 Hz, 2H), 6.81 (d, J = 8.3 Hz, 2H), 6.68 (d, J = 7.6 Hz, 1H), 5.34 (dd, J = 84.4, 15.3 Hz, 2H), 4.98 (s, 1H), 3.67 (s, 2H). $^{13}$C NMR $CDCl_3$ δ 157.45, 155.89, 138.47, 138.18, 133.10, 130.32, 129.15, 128.96, 128.88, 128.66, 128.53, 127.69, 127.50, 119.42, 116.54,

114.63, 55.82, 51.24, 51.19. Anal. Calcd for $C_{22}H_{21}N_5O$: C, 71.14; H, 5.70; N, 18.85. Found: C, 70.98; H, 5.69; N, 18.81.

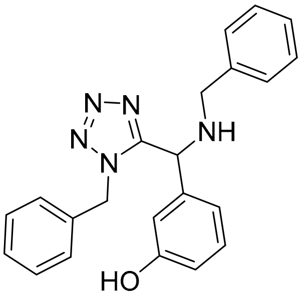


**Figure S1.** Structure of compound **AB15**.

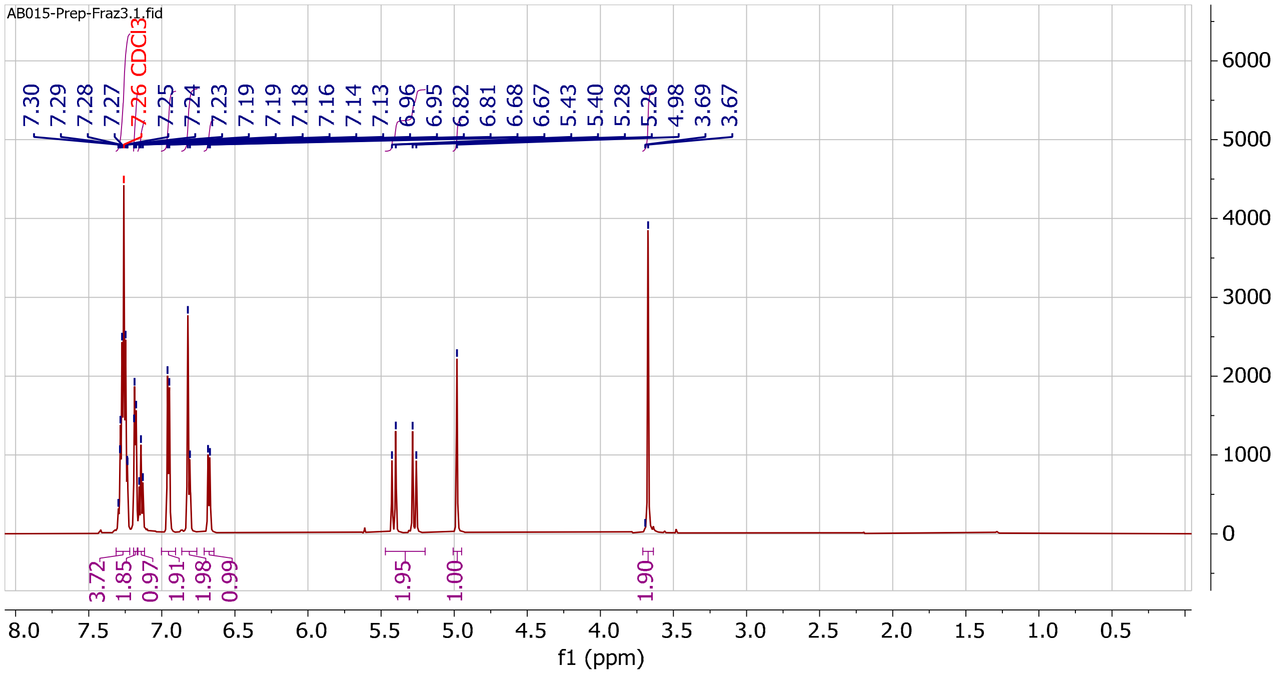


**Figure S2.** $^{1}$H NMR spectrum of compound **AB15**.

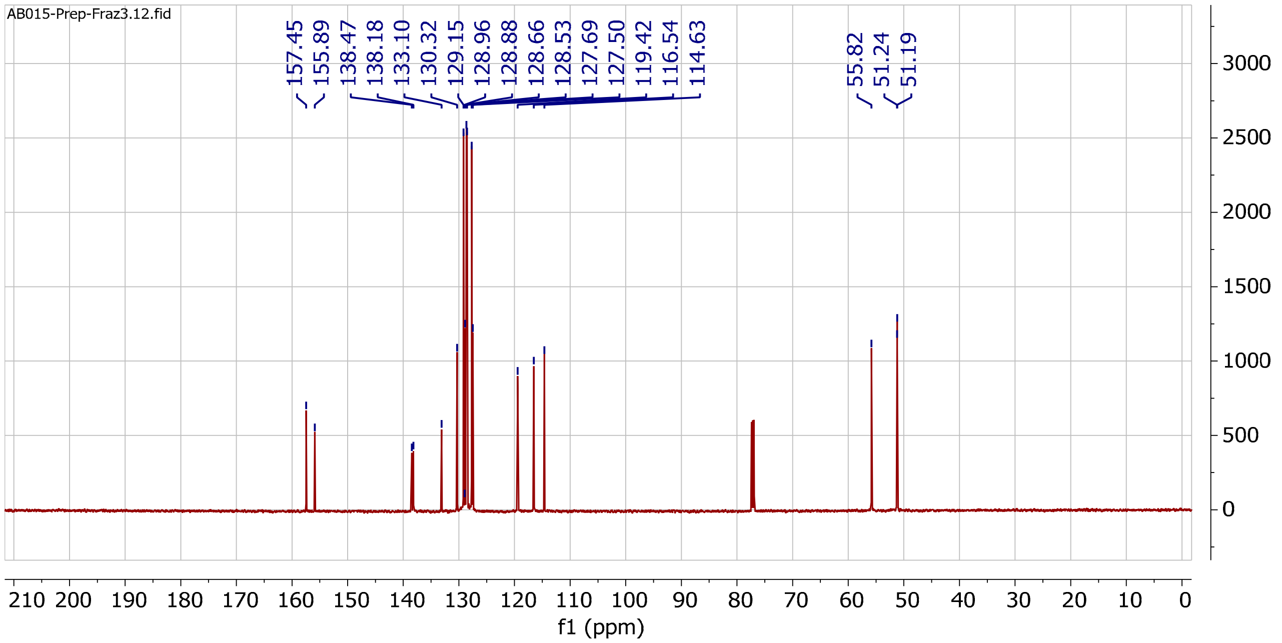


**Figure S3.** $^{13}C$ NMR spectrum of compound **AB15**.

*4-((1-benzyl-1H-tetrazol-5-yl)(benzylamino)methyl)phenol (**AB016**)*

The title compound prepared following the general procedure 1, was obtained as a white solid, after 5 days. $^{1}H$ NMR $CDCl_3$ δ 7.39 – 7.11 (m, 8H), 7.06 – 6.97 (m, 2H), 6.95 (d, J = 7.5 Hz, 2H), 6.81 – 6.69 (m, 2H), 5.34 (dd, J = 88.7, 15.3 Hz, 2H), 4.95 (s, 1H), 3.69 (s, 2H). $^{13}C$ NMR $CDCl_3$ δ 156.91, 156.85, 156.11, 156.08, 138.43, 133.13, 129.18, 129.01, 128.90, 128.71, 128.56, 127.63, 127.57, 116.39, 116.37, 55.44, 51.17. Anal. Calcd for $C_{22}H_{21}N_5O$: C, 71.14; H, 5.70; N, 18.85. Found: C, 71.00; H, 5.68; N, 18.80.

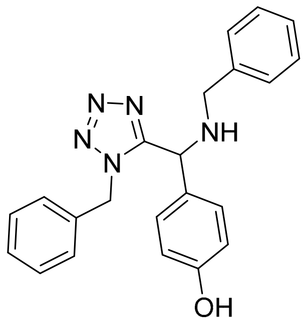


**Figure S4.** Structure of compound **AB16**.

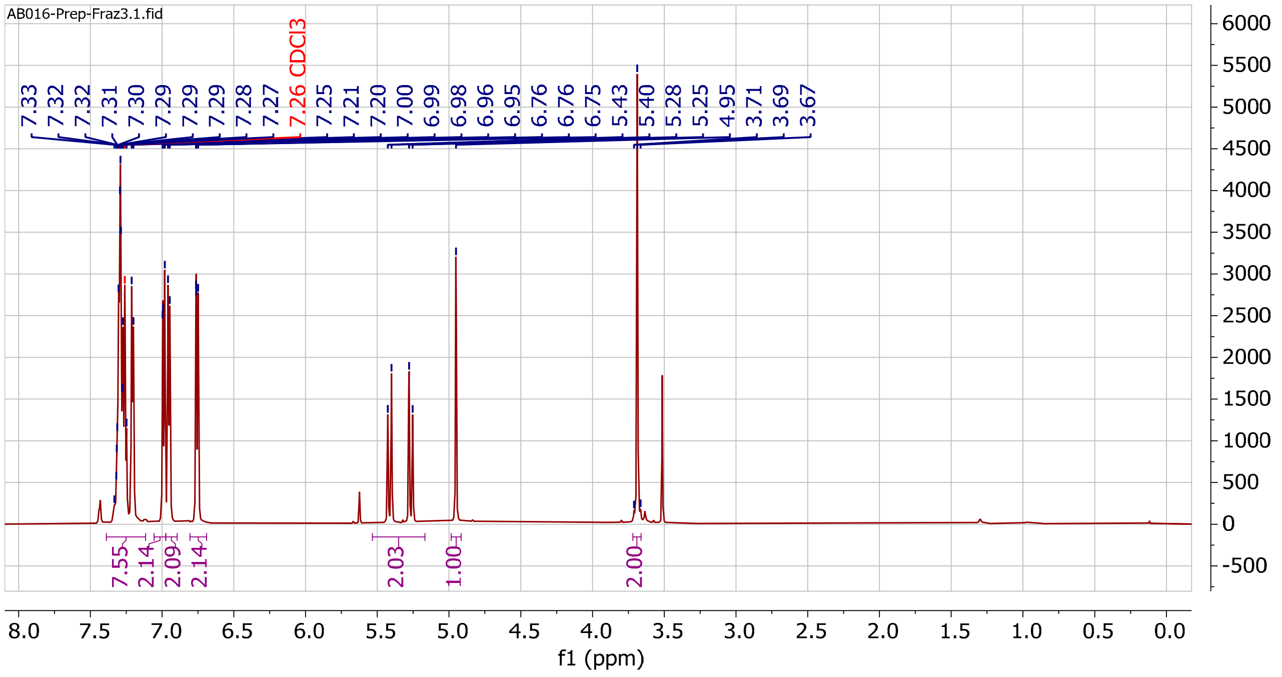


**Figure S5.** $^{1}$H NMR spectrum of compound **AB16**.

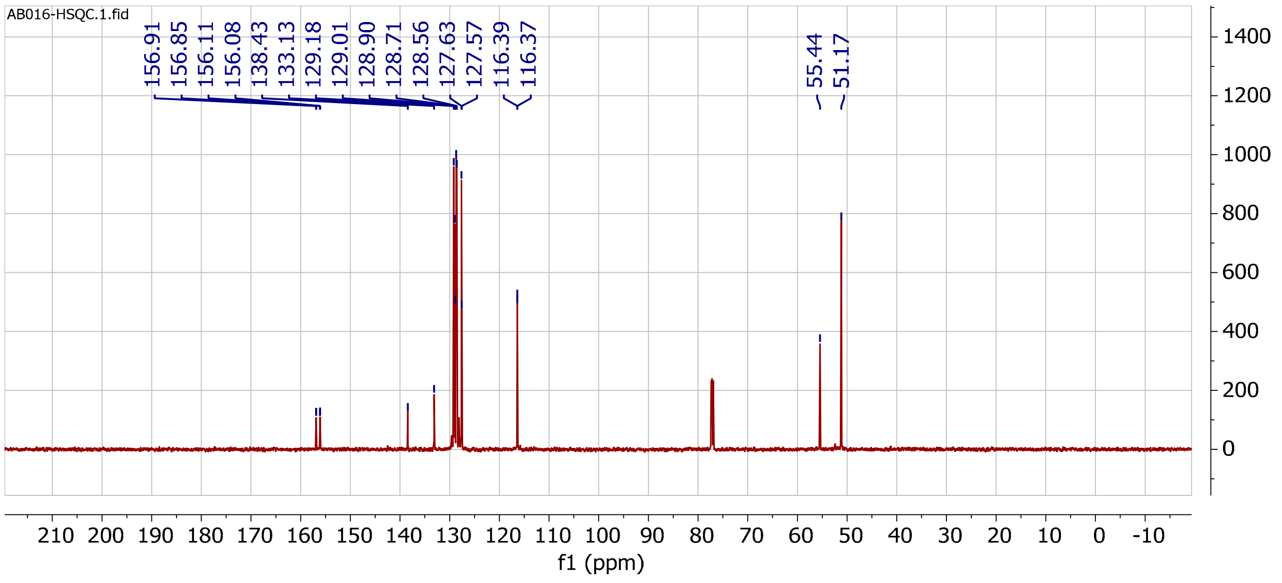


**Figure S6.** $^{13}$C NMR spectrum of compound **AB16**.

*N-benzyl-1-(1-benzyl-1H-tetrazol-5-yl)-1-phenylmethanamine (**AB017**)*

The title compound prepared following the general procedure 1, was obtained as a yellow oil, in 80% yield after 5 days. $^{1}H$ NMR $CDCl_3$ δ 7.30 – 7.26 (m, 4H), 7.25 (t, J = 7.2 Hz, 2H), 7.22 – 7.17 (m, 5H), 7.03 – 6.79 (m, 2H), 5.40 – 5.25 (m, 2H), 5.03 (s, 1H), 3.65 (s, 2H), 2.42 (s, 1H). $^{13}C$ NMR $CDCl_3$ δ 155.69, 138.71, 137.26, 133.23, 129.01, 128.95, 128.63, 128.57, 128.52, 128.32, 127.55, 127.44, 127.34, 55.87, 51.11, 50.90. Anal. Calcd for $C_{22}H_{21}N_5$: C, 74.34; H, 5.96; N, 19.70. Found: C, 74.16; H, 5.94; N, 19.66.

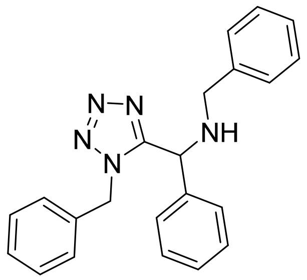


**Figure S7.** Structure of compound **AB17**.

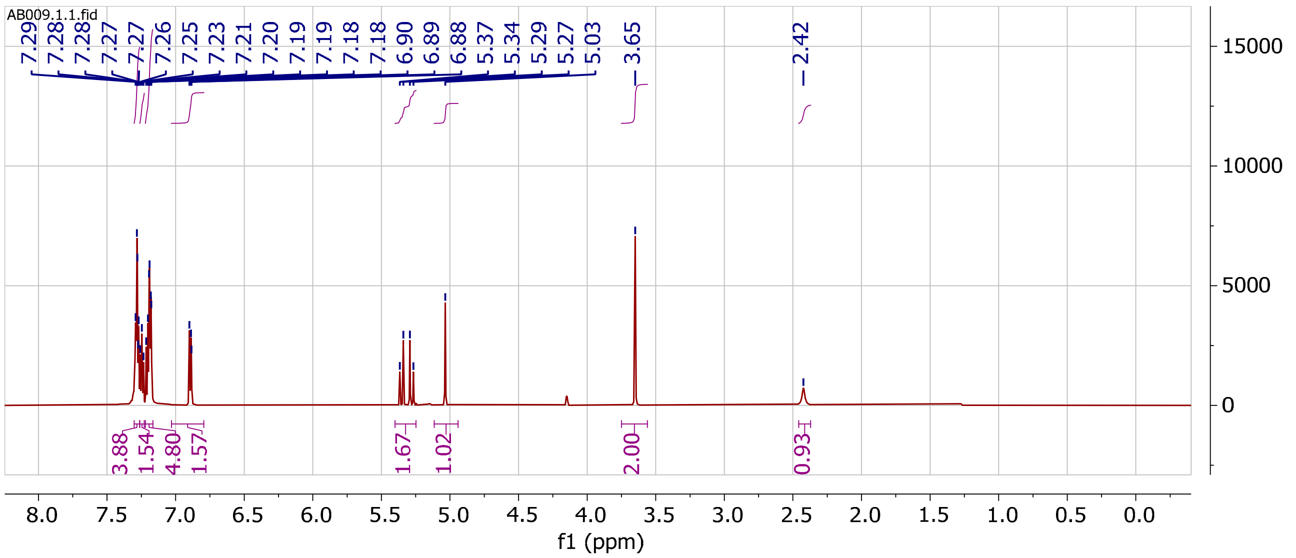


**Figure S8.** $^{1}H$ NMR spectrum of compound **AB17**.

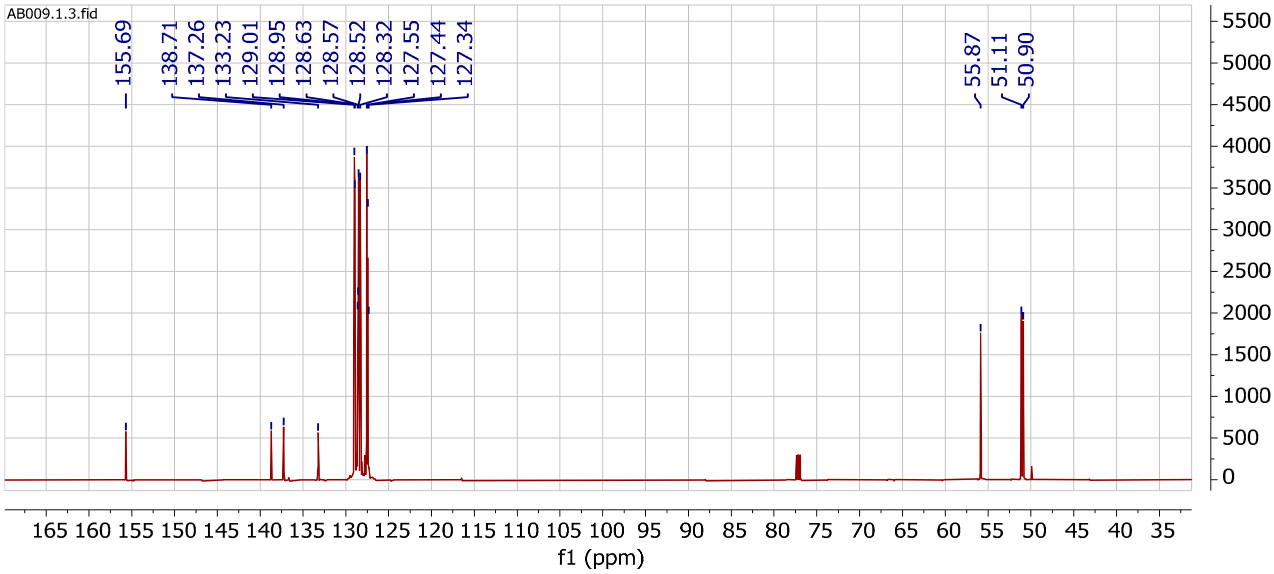


**Figure S9.** $^{13}C$ NMR spectrum of compound **AB17**.

*N-((1-benzyl-1H-tetrazol-5-yl)(phenyl)methyl)aniline (**AB027**)*

The title compound prepared following the general procedure 1, was obtained as a white solid, in 74% yield after 5 days. $^{1}H$ NMR $CDCl_3$ δ 7.38 – 7.30 (m, 7H), 7.23 (dd, J = 6.8, 2.9 Hz, 2H), 7.11 – 7.03 (m, 4H), 6.72 (t, J = 7.4 Hz, 1H), 6.39 (d, J = 8.0 Hz, 2H), 5.76 (s, 1H), 5.43 (dd, J = 178.5, 15.4 Hz, 2H), 4.65 (s, 1H). $^{13}C$ NMR $CDCl_3$ δ 155.54, 145.54, 136.90, 133.12, 129.41, 129.39, 129.37, 129.15, 129.03, 127.73, 127.48, 119.26, 113.86, 53.49, 51.37. Anal. Calcd for $C_{21}H_{19}N_5$: C, 73.88; H, 5.61; N, 20.51. Found: C, 73.68; H, 5.60; N, 20.47.

**Compound AB027**

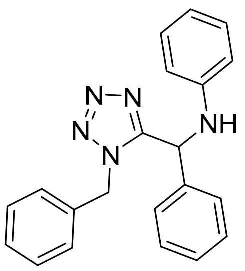


**Figure S10.** Structure of compound **AB27**.

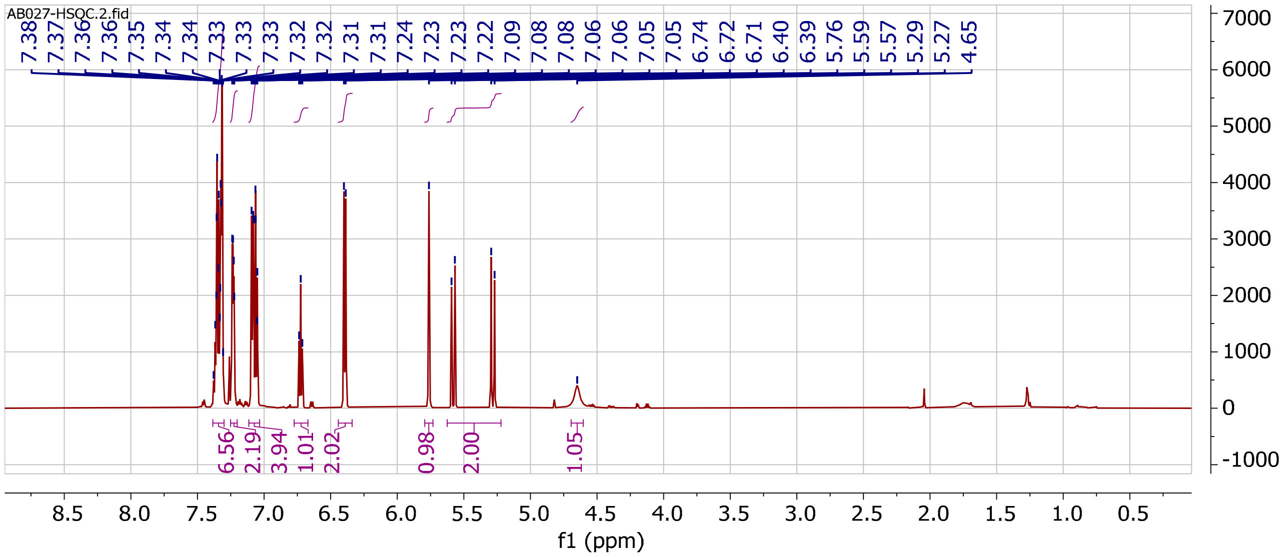


**Figure S11.** $^{1}$H NMR spectrum of compound **AB27**.

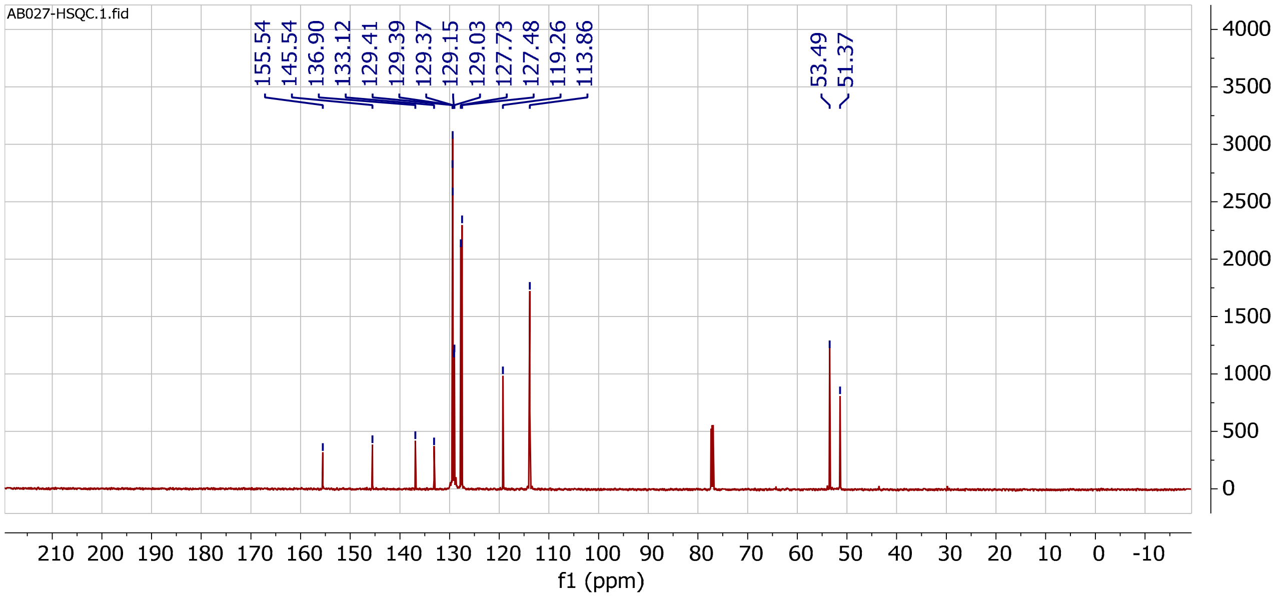


**Figure S12.** $^{13}$C NMR spectrum of compound **AB27**.

*3-((1-benzyl-1H-tetrazol-5-yl)(phenylamino)methyl)phenol (**AB028**)*

The title compound prepared following the general procedure 1, was obtained as a white solid, in 77% yield after 36 hours. $^{1}H$ NMR DMSO-$d_6$ δ 9.47 (d, J = 1.9 Hz, 1H), 7.31 – 7.27 (m, 3H), 7.15 – 7.08 (m, 3H), 7.05 – 7.00 (m, 2H), 6.86 – 6.81 (m, 2H), 6.68 (dd, J = 8.2, 2.3 Hz, 1H), 6.64 (d, J = 8.1 Hz, 3H), 6.58 (t, J = 7.3 Hz, 1H), 6.15 (d, J = 8.3 Hz, 1H), 5.77 – 5.61 (m, 2H). $^{13}C$ NMR DMSO-$d_6$ δ 157.59, 156.00, 146.68, 139.25, 134.20, 129.59, 128.80, 128.65, 128.32, 128.17, 118.38, 117.21, 115.12, 114.59, 113.26, 50.82, 50.25. Anal. Calcd for $C_{21}H_{19}N_5O$: C, 70.57; H, 5.36; N, 19.59. Found: C, 70.31; H, 5.34; N, 19.55.

**Compound AB028**

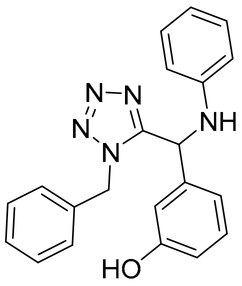


**Figure S13.** Structure of compound **AB28**.

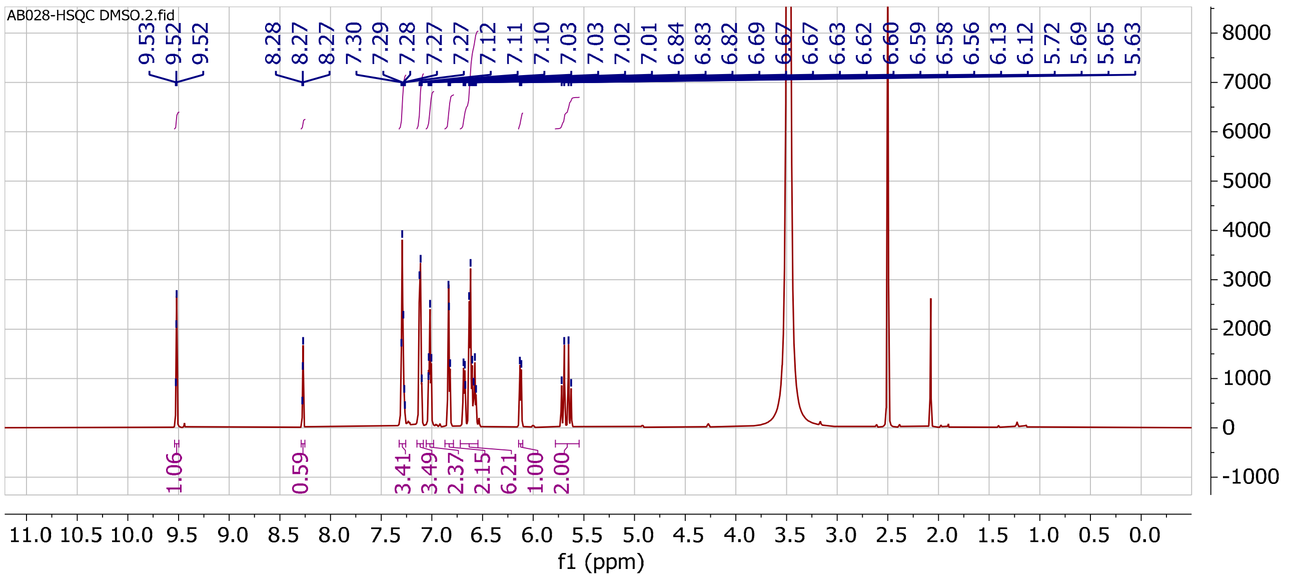


**Figure S14.** $^{1}H$ NMR spectrum of compound **AB28**.

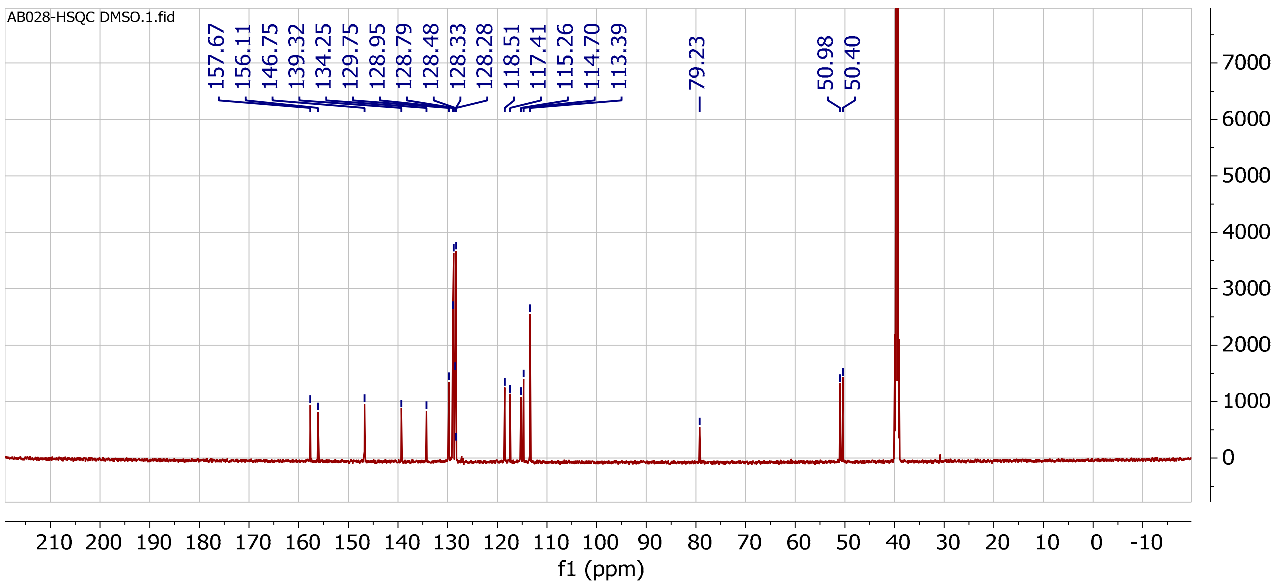


**Figure S15.** $^{13}C$ NMR spectrum of compound **AB28**.

*4-((1-benzyl-1H-tetrazol-5-yl)(phenylamino)methyl)phenol (**AB029**)*

The title compound prepared following the general procedure 1, was obtained as a white solid, in 63% yield after 36 hours. $^{1}H$ NMR $CD_3OD$ δ 7.29 (ddd, J = 9.0, 7.5, 6.1 Hz, 3H), 7.10 (dd, J = 10.1, 7.4 Hz, 5H), 7.02 (td, J = 7.8, 7.2, 1.4 Hz, 2H), 6.74 – 6.70 (m, 2H), 6.67 – 6.60 (m, 1H), 6.51 – 6.42 (m, 2H), 5.86 (s, 1H), 5.59 (dd, J = 127.4, 15.3 Hz, 2H). $^{13}C$ NMR $CD_3OD$ δ 158.96, 157.88, 147.81, 135.01, 130.11, 130.02, 129.98, 129.87, 129.68, 129.14, 129.03, 119.33, 116.78, 114.77, 53.77, 52.15. Anal. Calcd for $C_{21}H_{19}N_5O$: C, 70.57; H, 5.36; N, 19.59. Found: C, 70.38; H, 5.35; N, 19.54.

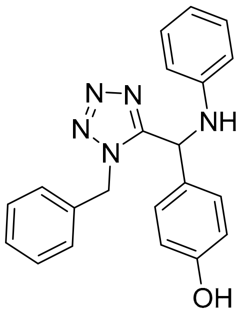


**Figure S16.** Structure of compound **AB29**.

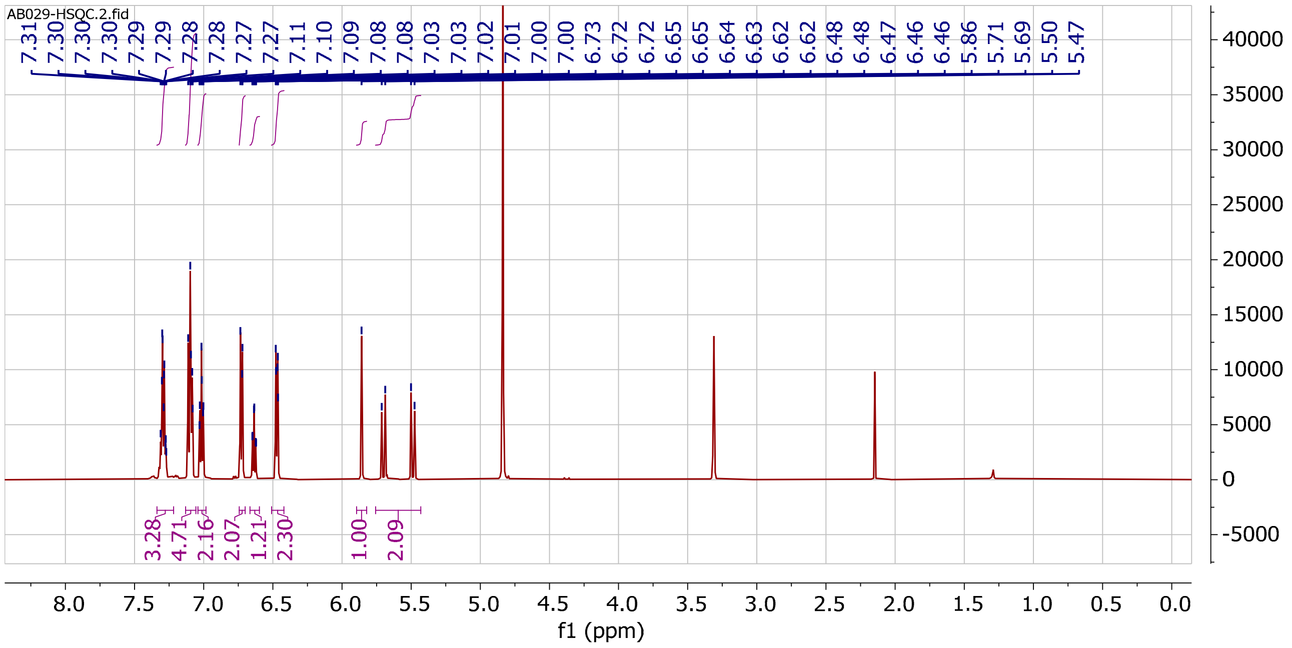


**Figure S17.** $^{1}$H NMR spectrum of compound **AB29**.

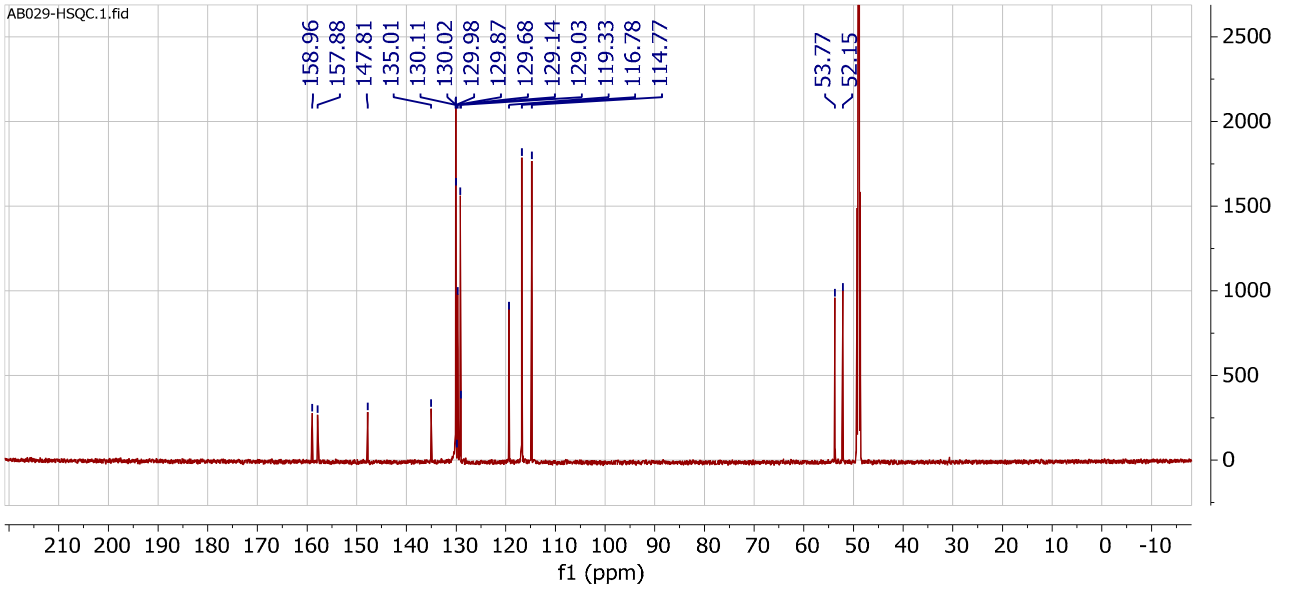


**Figure S18.** $^{13}$C NMR spectrum of compound **AB29**.

*Sodium 4-(((1-(4-(6,7-dimethoxy-3,4-dihydroisoquinolin-2(1H)-yl)-4-oxobutyl)-1H-tetrazol-5-yl)(thiophen-2-yl)methyl)amino)-3-hydroxybutanoate (**AD158**)*

The title compound was prepared following the general procedure 2. Yield MCR 27%, hydrolysis 97%, light-yellow oil, mixture of atropoisomers. $^1$H NMR $CD_3OD$ δ 7.42−7.35 (m, 1Ha,b), 7.15−7.07 (m, 1Ha,b), 7.00−6.94 (m, 1Ha,b), 6.75 (bs, 2Ha,b), 5.77 (bs, 1Ha,b), 4.61-4.49 (m, 4Ha,b), 4.14-4.05 (m, 1Ha,b), 3.80 (s, 6Ha,b), 3.74 (t, J=6 Hz, 2Ha), 3.60 (t, J=6 Hz, 2Hb), 2.80 (t, J=6 Hz, 2Hb), 2.74 (t, J=6 Hz, 2Ha), 2.72-2.56 (m, 2Ha,b), 2.51-2.32 (m, 4Ha,b), 2.18-2.06 (m, 1Ha,b). $^{13}$C NMR $CD_3OD$ δ 172.59, 172.51, 157.38, 149.41, 149.30, 149.24, 149.19, 142.77, 142.57, 128.22, 128.12, 127.97, 127.77, 127.68, 127.61, 126.43, 125.92, 113.02, 112.90, 110.95, 110.79, 69.14, 68.95, 64.99, 56.55, 56.51, 56.47, 56.44, 53.84, 53.69, 53.55, 48.16, 47.83, 45.04, 44.47, 42.20, 41.28, 30.78, 30.36, 29.67, 28.85, 25.86, 25.81. Anal. Calcd for $C_{25}H_{31}N_6NaO_6S$: C, 52.99; H, 5.51; N, 14.83. Found: C, 52.83; H, 5.50; N, 14.80.

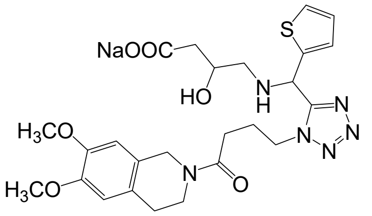


**5ce**

**Figure S19.** Structure of compound **AD158**.

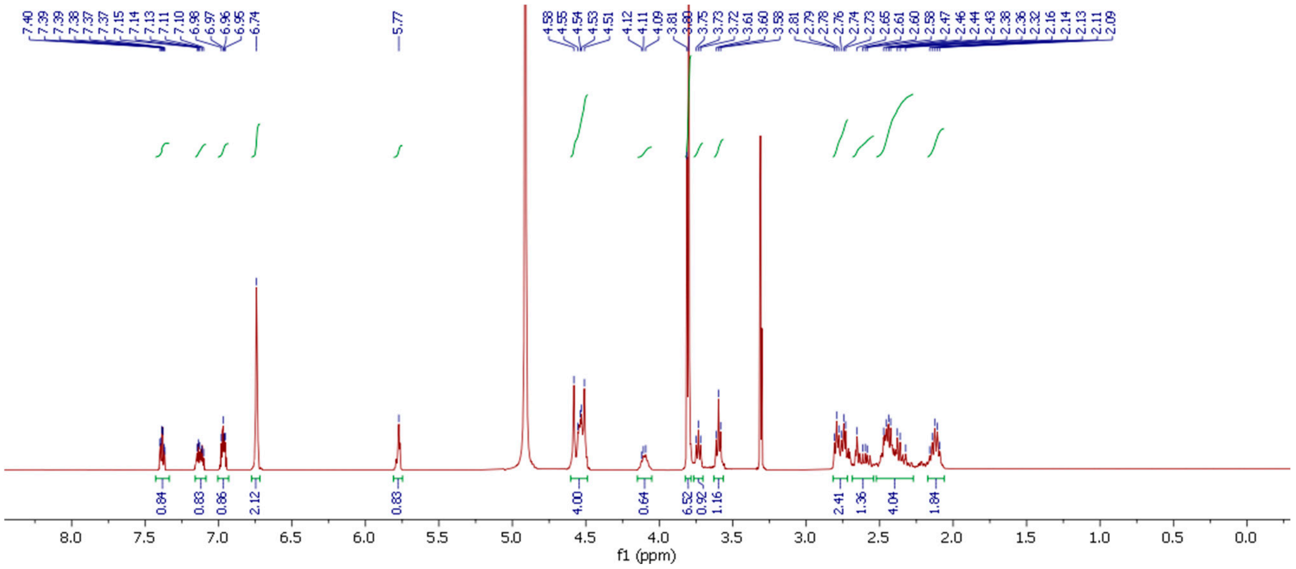


**Figure S20.** $^1$H NMR spectrum of compound **AD158**.

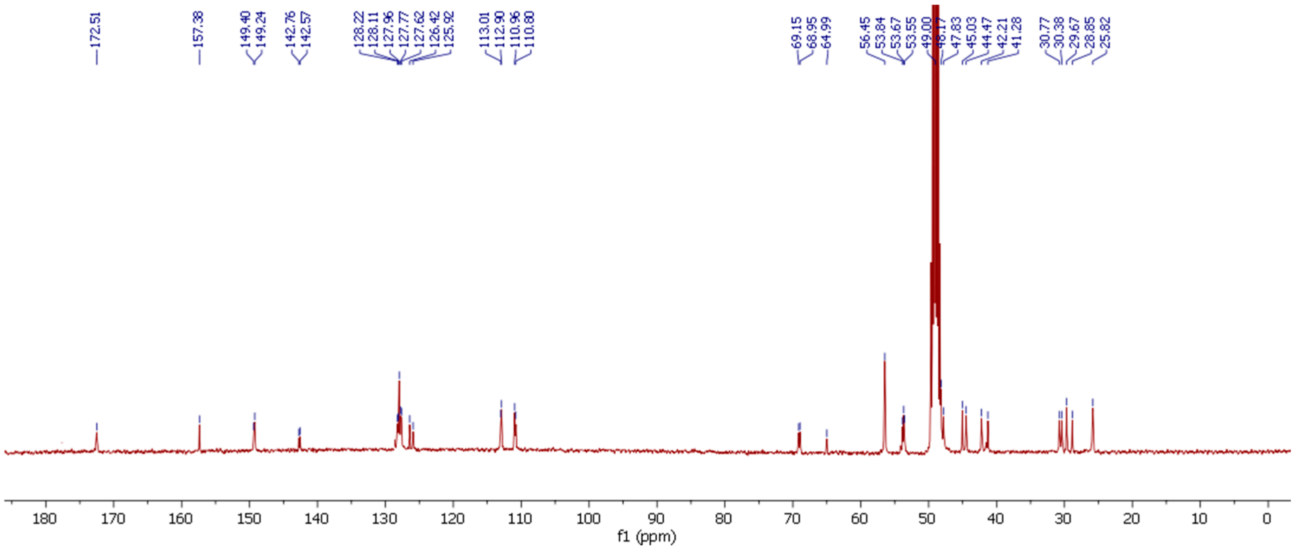


**Figure S21.** $^{13}C$ NMR spectrum of compound **AD158**.

*Sodium (3S)-4-(((1-(4-(3,4-dihydroisoquinolin-2(1H)-yl)-4-oxobutyl)-1H-tetrazol-5-yl)(thiophen-2-yl)methyl)amino)-3-hydroxybutanoate (**AD201**)*

The title compound was prepared following the general procedure 2. Yield MCR 27%, hydrolysis 69%, thick light-yellow oil, mixture of atropoisomers. $^{1}H$ NMR $CD_3OD$ δ 7.39−7.35 (m, 1Ha,b), 7.21−7.11 (m, 5Ha,b), 6.98−6.94 (m, 1Ha,b), 5.78 (s, 1Ha), 5.77 (s, 1Hb), 4.65 (bs, 2Ha), 4.58 (bs, 2Hb), 4.57-4.51 (m, 2Ha,b), 4.10-4.02 (m, 1Ha,b), 3.75 (t, J=5.6 Hz, 2Hb), 3.62 (t, J=5.6 Hz, 2Ha), 2.88 (t, J=6 Hz, 2Ha), 2.83 (t, J=6 Hz, 2Hb), 2.74-2.54 (m, 2Ha,b), 2.49-2.42 (m, 2Ha,b), 2.34-2.27 (m, 2Ha,b), 2.16-2.08 (m, 2Ha,b). $^{13}C$ NMR $CD_3OD$ δ 179.93, 172.67, 157.47, 142.95, 142.73, 136.19, 135.80, 134.46, 134.01, 129.61, 129.38, 127.98, 127.93, 127.84, 127.75, 127.57, 127.47, 127.32, 69.74, 69.49, 53.97, 53.90, 53.75, 48.23, 48.15, 45.36, 44.45, 43.29, 41.34, 30.82, 30.52, 30.18, 29.38, 25.80. Anal. Calcd for $C_{23}H_{27}N_6NaO_4S$: C, 54.53; H, 5.37; N, 16.59. Found: C, 54.44; H, 5.36; N, 16.57.

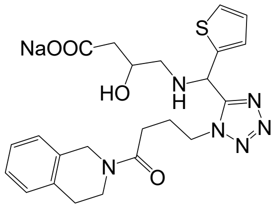


**5ah**

**Figure S22.** Structure of compound **AD201**.

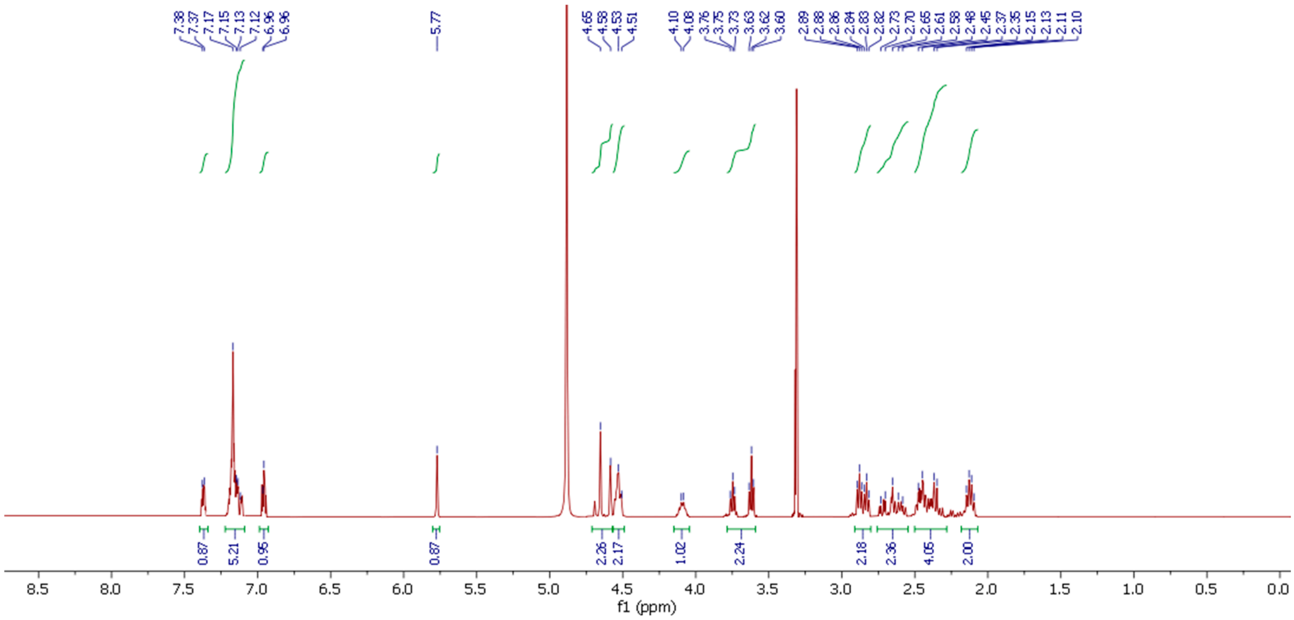

**Figure S23.** $^{1}$H NMR spectrum of compound **AD201**.

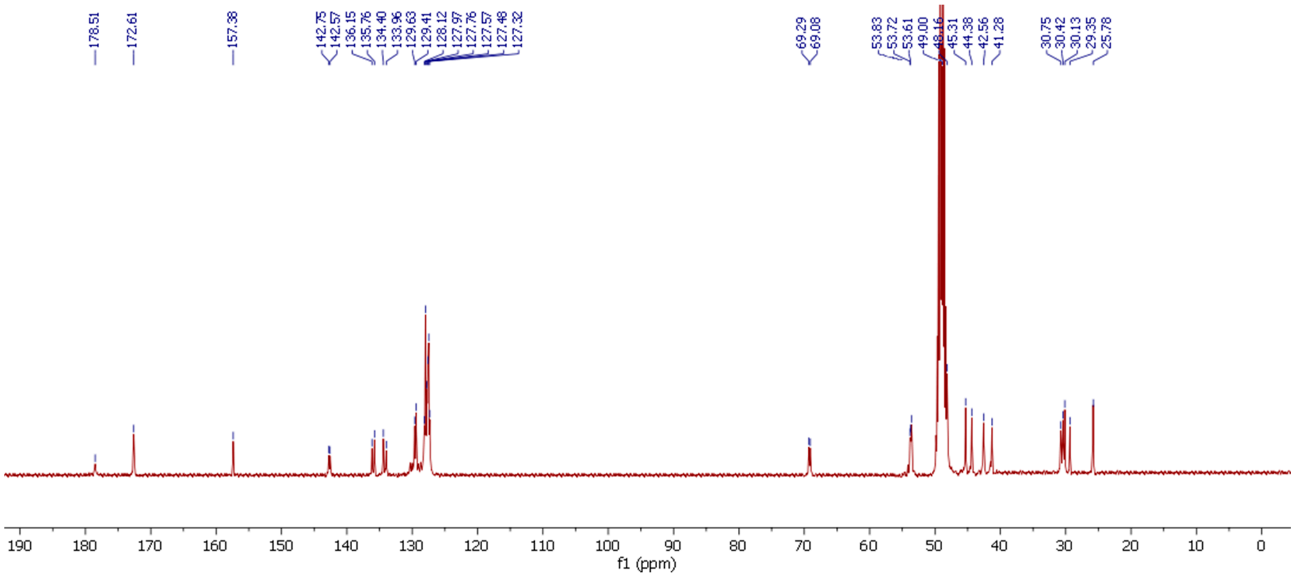

**Figure S24.** $^{13}$C NMR spectrum of compound **AD201**.

*Sodium (3S)-4-(((1-(4-(3,4-dihydroisoquinolin-2(1H)-yl)-4-oxobutyl)-1H-tetrazol-5-yl)(phenyl)methyl)amino)-3-hydroxybutanoate (**AD202**)*

The title compound was prepared following the general procedure 2. Yield MCR 45%, hydrolysis 80%, thick light-yellow oil, mixture of atropoisomers. $^1$H NMR $CD_3OD$ δ 7.47−7.26 (m, 5Ha,b), 7.21−7.13 (m, 4Ha,b), 5.42 (s, 1Ha,b), 4.65 (bs, 2Ha), 4.55 (bs, 2Hb), 4.49-4.40 (m, 2Ha,b), 4.15-4.05 (m, 1Ha,b), 3.76-3.71 (m, 2Hb), 3.60-3.55 (m, 2Ha), 2.87 (t, J=6 Hz, 2Ha), 2.83 (t, J=6.4 Hz, 2Hb), 2.72-2.53 (m, 2Ha,b), 2.45-2.30 (m, 4Ha,b), 2.09-1.98 (m, 2Ha,b). $^{13}$C NMR $CD_3OD$ δ 180.15, 180.10, 172.57, 157.79, 157.76, 139.40, 139.24, 136.15, 135.74, 134.41, 133.94, 130.11, 129.63, 129.38, 128.96, 127.97, 127.75, 127.56, 127.44, 127.28, 69.62, 69.47, 58.39, 58.18, 54.22, 53.97, 48.09, 47.99, 45.30, 44.37, 43.39, 43.34, 41.28, 30.73, 30.43, 30.14, 29.35, 25.68. Anal. Calcd for $C_{25}H_{29}N_6NaO_4$: C, 59.99; H, 5.84; N, 16.79. Found: C, 59.81; H, 5.83; N, 16.78.

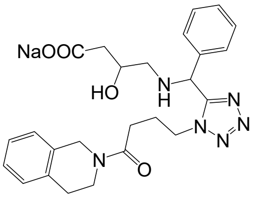


**5ag**

**Figure S25.** Structure of compound **AD202**.

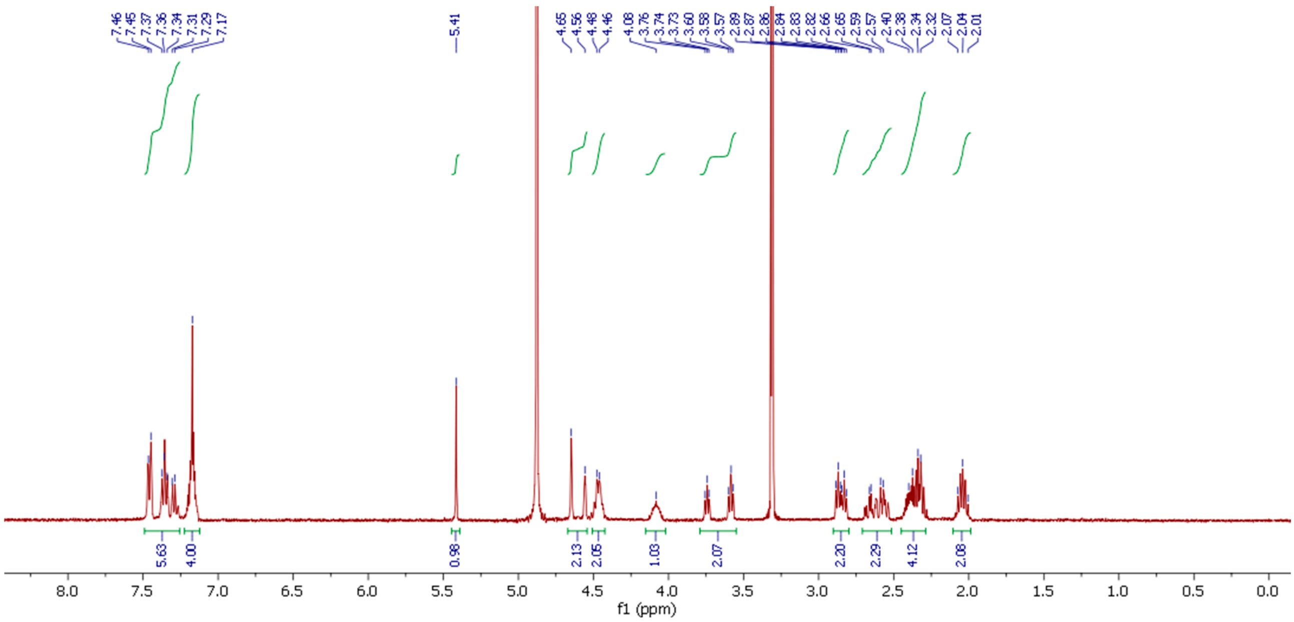


**Figure S26.** $^1$H NMR spectrum of compound **AD202**.

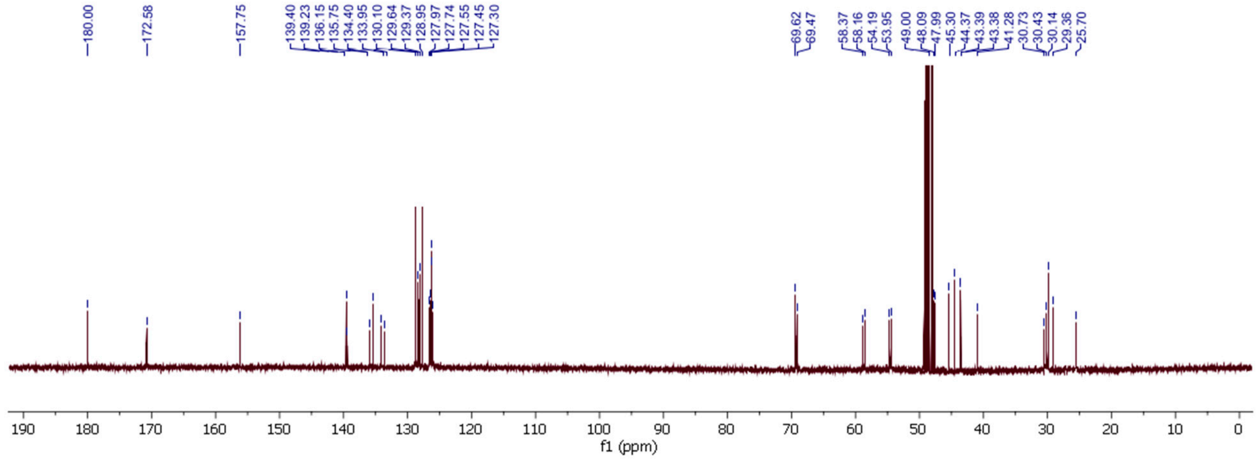


**Figure S27.** $^{13}C$ NMR spectrum of compound **AD202**.

*Sodium (3S)-4-(((1-(4-(benzylamino)-4-oxobutyl)-1H-tetrazol-5-yl)(thiophen-2-yl)methyl)amino)-3-hydroxybutanoate (**AD205**)*

The title compound was prepared following the general procedure 2. Yield MCR 39%, hydrolysis 88%, light-yellow oil, mixture of atropoisomers. $^{1}H$ NMR $CD_3OD$ δ 7.40 (dd, J=2, 1.2 Hz, 1Ha), 7.39 (dd, J=2, 1.2 Hz, 1Hb), 7.34-7.21 (m, 5Ha,b), 7.13-7.11 (m, 1Ha), 7.11-7.08 (m, 1Hb), 6.99 (dd, J=3.6, 2.8 Hz, 1Ha), 6.97 (dd, J=3.6, 2.8 Hz, 1Hb), 5.74 (s, 1Ha), 5.73 (s, 1Hb), 4.53-4.47 (m, 2Ha,b), 4.35 (s, 2Ha,b), 4.09-4.01 (m, 1Ha,b), 2.71(dd, J=12, 4 Hz, 1Ha), 2.65-2.62 (m, 1Ha,b), 2.56 (dd, J=12.4, 7.6 Hz, 1Hb), 2.34-2.25 (m, 4Ha,b), 2.12-2.04 (m, 2Ha,b). $^{13}C$ NMR $CD_3OD$ δ 180.15, 180.10, 174.09, 157.38, 157.36, 142.77, 142.59, 139.99, 129.54, 128.62, 128.19, 127.97, 127.96, 127.82, 127.52, 127.48, 69.70, 69.50, 54.03, 53.90, 53.87, 53.77, 48.23, 44.15, 43.38, 43.34, 33.28, 26.39. Anal. Calcd for $C_{21}H_{25}N_6NaO_4S$: C, 52.49; H, 5.24; N, 17.49. Found: C, 52.38; H, 5.21; N, 17.47.

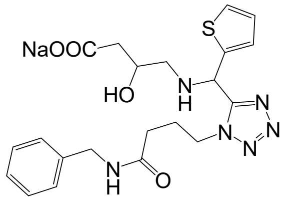


**5bf**

**Figure S28.** Structure of compound **AD205**.

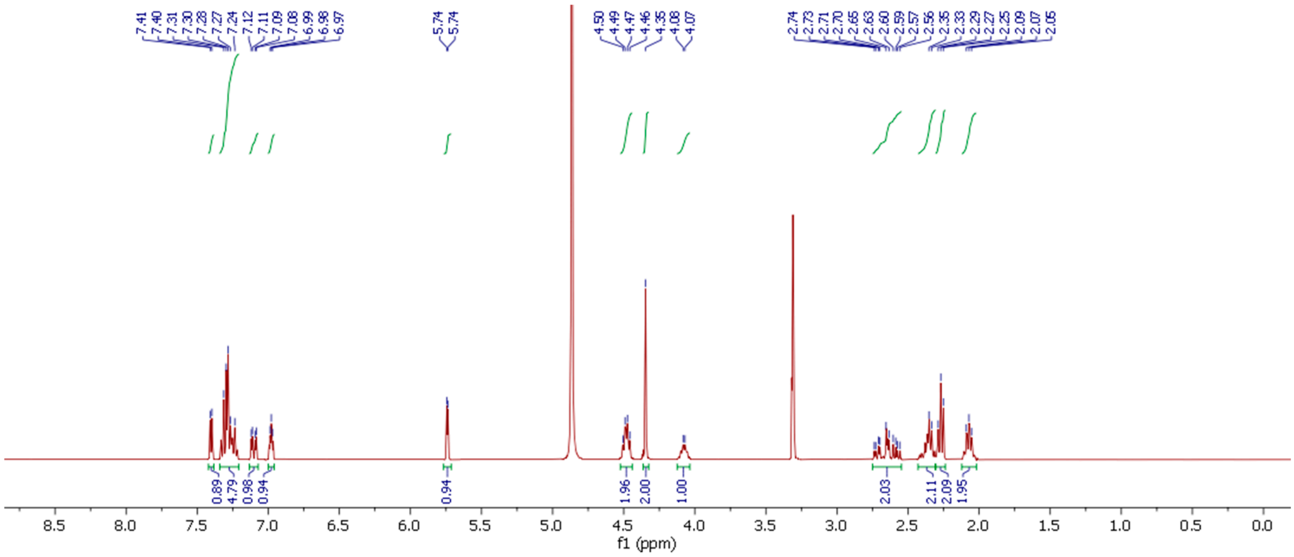


**Figure S29.** $^{1}$H NMR spectrum of compound **AD205.**

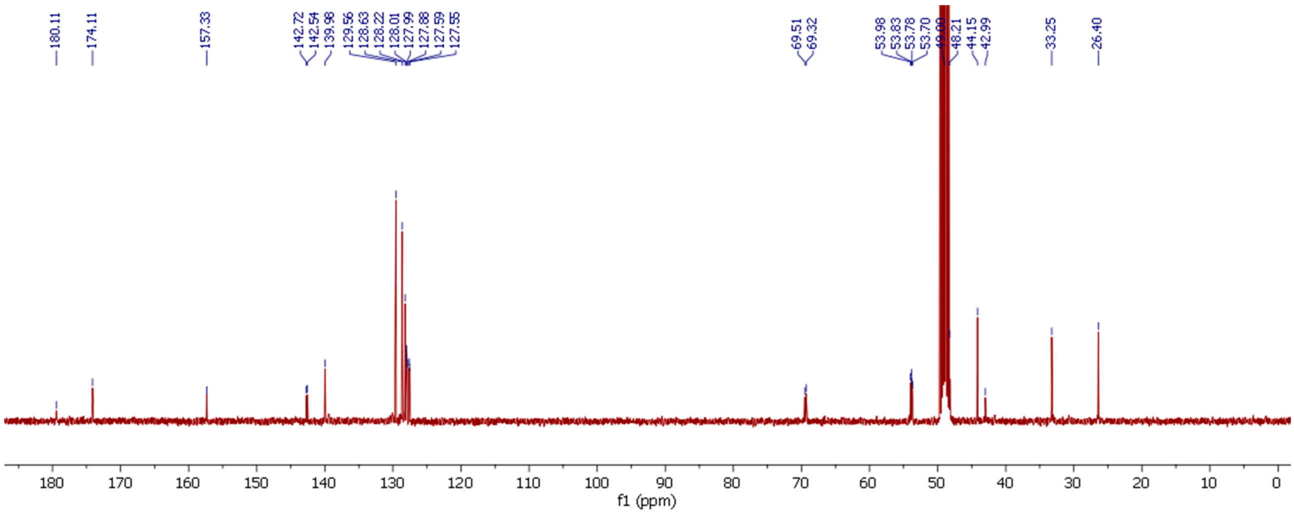


**Figure S30.** $^{13}$C NMR spectrum of compound **AD205**.

*Sodium (3S)-4-(((1-(4-(benzylamino)-4-oxobutyl)-1H-tetrazol-5-yl)(phenyl)methyl)amino)-3-hydroxybutanoate (**AD206**)*

The title compound was prepared following the general procedure 2. Yield MCR 45%, hydrolysis 64%, light-yellow oil, mixture of atropoisomers. $^1$H NMR $CD_3OD$ δ 7.44–7.22 (m, 10Ha,b), 5.38 (s, 1Ha), 5.37 (s, 1Hb), 4.42 (q, J=8 Hz, 2Ha,b), 4.34 (s, 2Ha,b), 4.11-4.03 (m, 1Ha,b), 2.69-2.52 (m, 2Ha,b), 2.32-2.28 (m, 2Ha,b), 2.25-2.20 (m, 2Ha,b), 2.03-1.94 (m, 2Ha,b). $^{13}$C NMR $CD_3OD$ δ 180.13, 180.06, 174.03, 157.69, 139.97, 139.31, 139.16, 130.11, 129.63, 129.54, 128.90, 128.60, 128.19, 69.61, 69.51, 58.43, 58.27, 54.15, 54.00, 48.00, 44.13, 43.44, 43.36, 33.21, 26.29. Anal. Calcd for $C_{23}H_{27}N_6NaO_4$: C, 58.22; H, 5.74; N, 17.71. Found: C, 58.08; H, 5.71; N, 17.69.

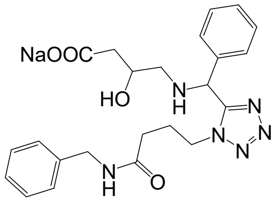


**5be**

**Figure S31.** Structure of compound **AD206**.

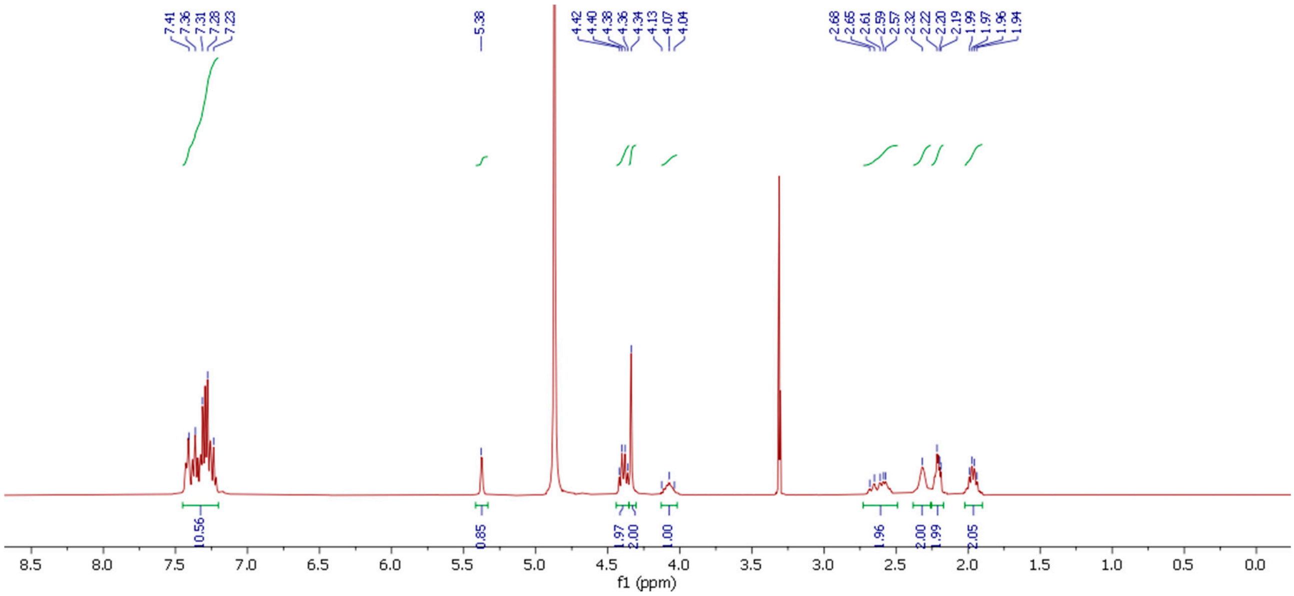


**Figure S32.** $^1$H NMR spectrum of compound **AD206.**

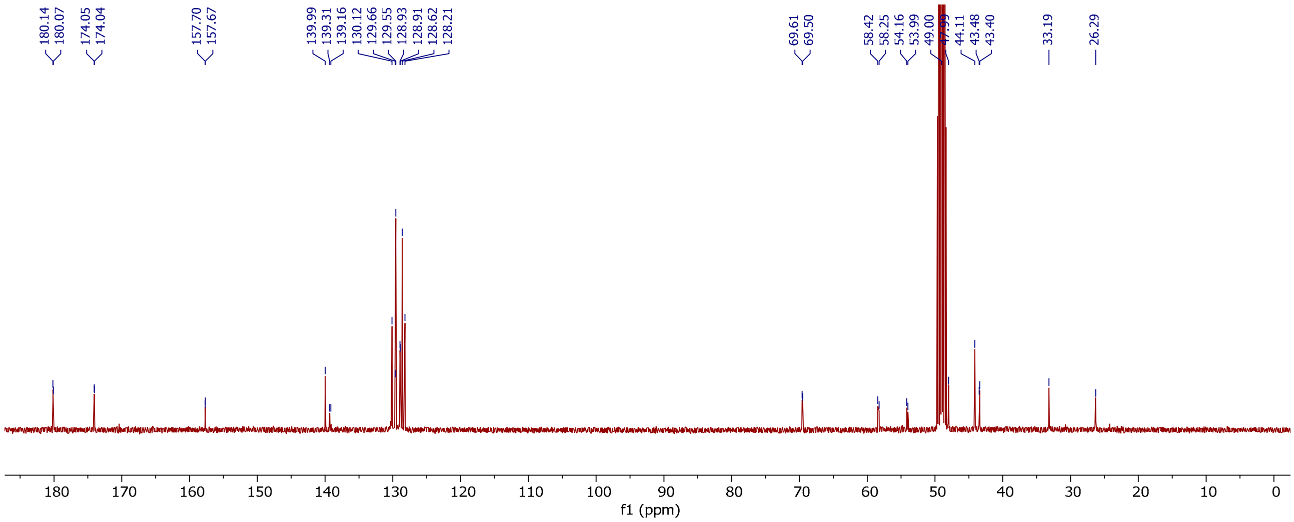


**Figure S33.** $^{13}C$ NMR spectrum of compound **AD206**.

## References

Fausta Ulgheri, Pietro Spanu, Francesco Deligia, Giovanni Loriga, Maria Pia Fuggetta, Iris de Haan, Ajay Chandgudge, Matthew Groves, Alexander Domling, Design, synthesis and biological evaluation of 1,5-disubstituted α-amino tetrazole derivatives as non-covalent inflammasome-caspase-1 complex inhibitors with potential application against immune and inflammatory disorders. *Eur. J. Med. Chem.* **2022**, *229*, 114002. doi.org/10.1016/j.ejmech.2021.114002

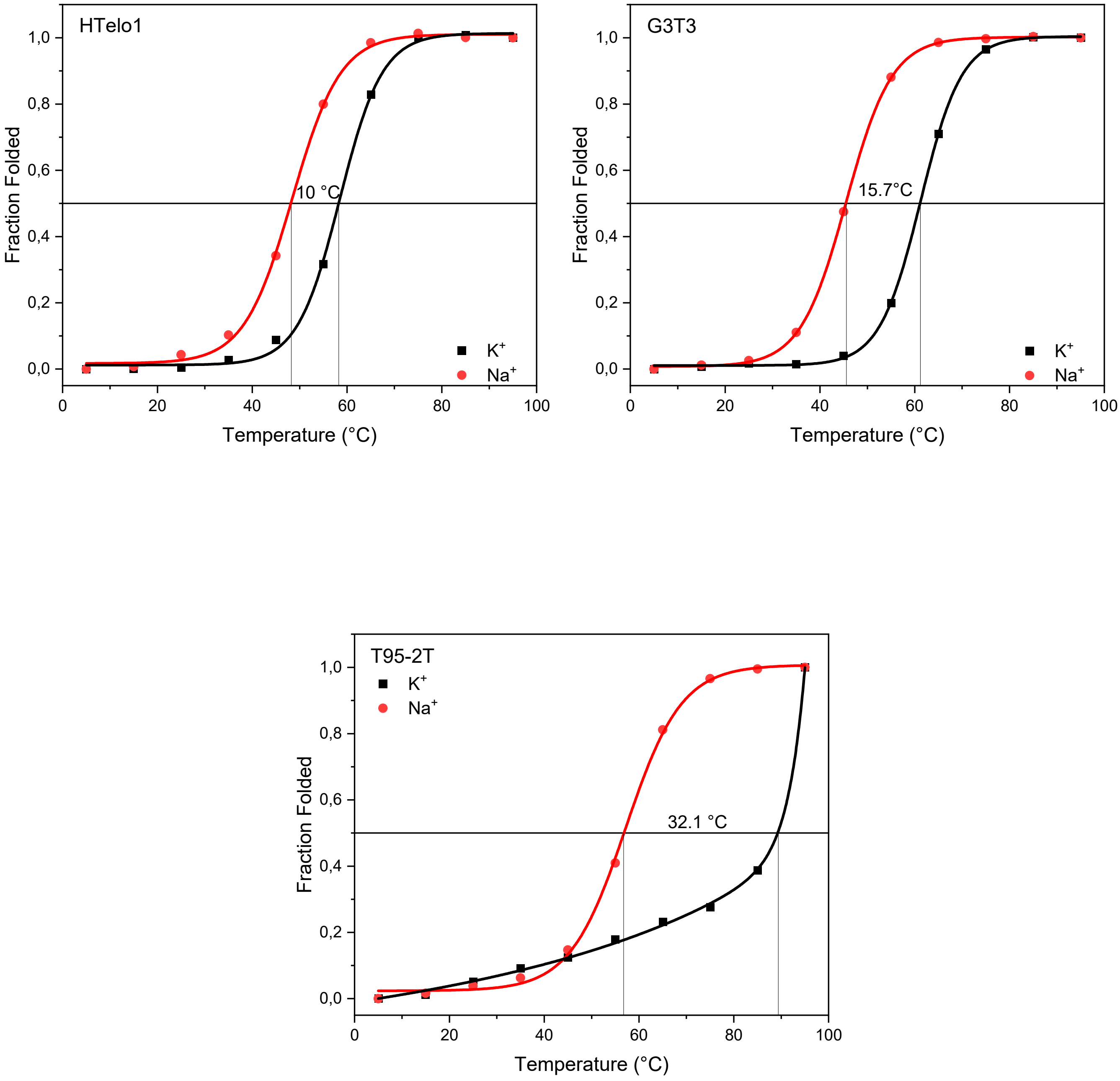


**Figure S34.** SRCD melting curves for HTelo 1, G3T3, and T95-2T G4s in 10 mM phosphate buffer, pH 7.4, in the presence of 70 mM potassium or sodium ions (indicated). The ellipticity was monitored at the maximum wavelength of each G4 spectrum.
